# Rice microtubule-associated protein OsMAP65-3.1, but not OsMAP65-3.2, plays a critical role in phragmoplast microtubule organization in cytokinesis

**DOI:** 10.3389/fpls.2022.1030247

**Published:** 2022-10-26

**Authors:** Xiaoli Lin, Yu Xiao, Yongping Song, Cong Gan, Xingguang Deng, Peng Wang, Jialong Liu, Zhishu Jiang, Limei Peng, Dahu Zhou, Xiaopeng He, Jianmin Bian, Changlan Zhu, Bo Liu, Haohua He, Jie Xu

**Affiliations:** ^1^ Key Laboratory of Crop Physiology, Ecology, and Genetic Breeding of the Ministry of Education, Jiangxi Agricultural University, Nanchang, Jiangxi, China; ^2^ Key Laboratory of Bio-Resource and Eco-Environment of Ministry of Education, College of Life Sciences, State Key Laboratory of Hydraulics and Mountain River Engineering, Sichuan University, Chengdu, Sichuan, China; ^3^ Department of Plant Biology, College of Biological Sciences, University of California, Davis, Davis, CA, United States

**Keywords:** rice, MAP65-3, cytokinesis, phragmoplast, MYB3R transcription factor

## Abstract

In plants, MAP65 preferentially cross-links the anti-parallel microtubules (MTs) and plays an important role for cytokinesis. However, the functions of MAP65 isoforms in rice (*Oryza sativa.* L) are largely unknown. Here, we identified two MAP65-3 homologs in rice, OsMAP65-3.1 and OsMAP65-3.2. We found that both OsMAP65-3.1 and OsMAP65-3.2 were similar in dimerization and location to AtMAP65-3, and the expression of either rice genes driven by the *AtMAP65-3* promoter suppressed the cytokinesis failure and growth defect of *atmap65-3*. However, *OsMAP65-3.1* with native promoter also recovered the *atmap65-3*, but *OsMAP65-3.2* with its own promoter had no effects. *OsMAP65-3.1* but not *OsMAP65-3.2* was actively expressed in tissues enriched with dividing cells. R1R2R3-Myb (MYB3R) transcription factors directly bound to the *OsMAP65-3.1* promoter but not that of *OsMAP65-3.2*. Furthermore, *osmap65-3.2* had no obvious phenotype, while either *osmap65-3.1* or *osmap65-3.1(+/-)* was lethal. The eminent MTs around the daughter nuclei and cytokinesis defects were frequently observed in *OsMAP65-3.1*-defective plants. Taken together, our findings suggest that *OsMAP65-3.1*, rather than *OsMAP65-3.2*, plays essential roles in rice cytokinesis resulting from their differential expression which were passably directly regulated by OsMYB3Rs.

## Introduction

Cytokinesis is the final step of cell division to divide the mother cell into two daughter cells and is of fundamental importance in both animals and plants. In animal cells, cytokinesis is normally completed through the formation of the cleavage furrow and contractile ring, without additional cell membrane biosynthesis (Glotzer, 2001). In contrast, the cell walls of plants thwart the invagination of the mother cell membrane; therefore, these cells generate a plant-specific membrane compartment, the cell plate, to facilitate the separation of daughter cells (Jürgens, 2005; McMichael and Bednarek, 2013). Plant cytokinesis results in the formation of the cell plate from the interior to the periphery of the cell; this process is executed by the phragmoplast, which mediates membrane-trafficking events delivering Golgi vesicles to the outspread cell plate (Smertenko et al., 2017; Smertenko et al., 2018).

The phragmoplast contains a bipolar array of highly dynamic microtubules (MTs), actin filaments, and endomembranes (Park et al., 2012; Smertenko et al., 2017). The minus ends of the phragmoplast MTs point toward the daughter nuclei, and the plus ends face each other adjoining the plane of cell division (Staehelin and Hepler, 1996). The phragmoplast MT array is a result of the polymerization, depolymerization, and reorganization of the spindle midzone MTs, with mixed polarities occurring during late anaphase and early telophase, before being organized into a solid bipolar array (Zhang et al., 1990). During cytokinesis, phragmoplast MTs begin to depolymerize at their center, where vesicles have already been fused to the cell plate, and polymerization at the periphery of the phragmoplast leads to the expansion of the array, giving rise to a ring-shaped structure (Lloyd, 2011; Murata et al., 2013; Müller and Jürgens, 2016).

A batch of regulatory proteins are required to sustain the bipolar configuration of phragmoplast MTs during their expansion (Hamada, 2014; Smertenko et al., 2018; Lee and Liu, 2019). Mutations of the genes encoding MT nucleation facotrs cause aberrant phragmoplast organization and aborted cytokinesis (Kong et al., 2010; Ho et al., 2011b; Hotta et al., 2012; Janski et al., 2012; Murata et al., 2013; Lee and Liu, 2019; Ma and Han, 2020). Several proteins with a locational preference for MT plus-ends facilitate the phragmoplast MT polymerization (Sedbrook et al., 2004; Kawamura et al., 2006; Ambrose et al., 2007; Bisgrove et al., 2008; Komaki et al., 2010). In addition, MT bundling is mediated by the dimerization of the MT-associated proteins (MAPs) in the MAP65 family that preferentially cross-link anti-parallel MTs (Müller et al., 2002; Müller et al., 2004; Ho et al., 2011a; Ho et al., 2011b; Ho et al., 2012; Murata et al., 2013). The MAP65 family in *Arabidopsis thaliana* contains nine members that show relatively conserved N-terminal halves and variable C-terminal halves (Smertenko et al., 2008). Because of their different localization and expression patterns, despite they share similar biochemical activity in MT bundling, they are thought to carry out different intracellular functions (Van Damme et al., 2004; Smertenko et al., 2008). MAP65-3 is a cytokinesis-specific MAP65 isoform and plays primary roles in phragmoplast integrity and efficient cell plate formation. MAP65-3 first emerges in the middle region of the central spindle after anaphase onset and, later in telophase, becomes gradually concentrated at the phragmoplast midzone (Müller et al., 2004; Van Damme et al., 2004; Smertenko et al., 2008; Ho et al., 2011a). Loss of *MAP65-3* leads to a wider phragmoplast midzone and frequent cytokinesis failures (incomplete cell plate or multiple-nuclei cells); the mutants also exhibit abnormal post-embryonic development and reduced fertility (Müller et al., 2004; Caillaud et al., 2008; Ho et al., 2011a).

Phragmoplast dynamics during cytokinesis are closely related to MAP65 activity, which is regulated by phosphorylation. Phosphorylation deactivates MAP65, leading to the debundling of MTs (Sasabe and Machida, 2012; Boruc et al., 2017). Mitogen-activated protein kinase NPK1 in tobacco (*Nicotiana tabacum*) is the first protein to phosphorylate NtMAP65-1 (Sasabe et al., 2006). The MPK4 enzyme in *Arabidopsis* has been demonstrated to phosphorylate several MAP65 proteins, including MAP65-3. *MPK4* mutants exhibit the abortive or delayed transition of mitotic and cytokinetic MTs and severely bundled MTs (Beck et al., 2010; Beck et al., 2011). Aurora kinase has also been shown to phosphorylate and deactivate MAP65 (Boruc et al., 2017). In addition, Zhang et al. (2018) reported a positive regulation mechanism of MAP65-3 activities in the phragmoplast. BUB3;1 and BUB3;2 proteins interact with MAP65-3 and promote MT bundling for phragmoplast expansion. Besides the aforementioned proteins, the MT motor protein kinesin-12 is critical for maintaining MT plus ends in the phragmoplast midzone (Lee et al., 2007). *Arabidopsis* double-mutants *pok1/pok2* (two kinesin-12 orthologs) show chaotic division sites and a slower phragmoplast expansion rate compared to wild-type (Herrmann et al., 2018).

Remarkable strides forward have been made in understanding mechanisms of plant mitosis and cytokinesis. Nearly all published studies have been carried out on *A. thaliana* and tobacco BY-2 cells, which are dicot systems. Rice (*Oryza sativa.* L) is the most important cereal crop and a model Graminaceae species used in molecular biology. However, a few if any studies have been devoted to understanding molecular mechanisms of cytokinesis during mitotic division in rice. Researching the function of MAP65-3 homologs is a good starting point for unraveling cytokinesis in rice. In this study, we dissected the functions of two rice MAP65-3 homologs, Os01g0685900 (OsMAP65-3.1) and Os05g0552900 (OsMAP65-3.2). We showed that both proteins are functional in cytokinesis, but only OsMAP65-3.1 plays essential roles in rice cytokinesis, as only this protein’s expression was activated in mitosis by positive regulators of late-cell-cycle genes (i.e., R1R2R3-Myb [MYB3R] transcription factors).

## Materials and methods

### Plant materials and growth conditions

All rice mutants used in this study (such the *osmap65-3.1* heterozygote, *osmap65-3.2*, *osmyb3r2*, and *osmyb3r2-l*) were generated with the CRISPR/Cas9 technique in a Japonica cv Nipponbare. All rice plants were grown in an experimental field of Jiangxi Agricultural University under local growing conditions in Nanchang, Jiangxi Province, China. In order to artistically and directly exhibit rice growth and development, plants were transplanted to pots for photographing in maturation stage. *Arabidopsis thaliana* materials, including the wild-type (Columbia-0) and *atmap65-3* (salk022166) plants, were grown under a 16 h light/8 h dark cycle with 70% relative humidity at 22°C. To collect root tips for immunolocalization, rice was grown in water-nutrient solution at 28 °C with a 14 h light/10 h dark cycle for 14 days, while *Arabidopsis* seeds were germinated on 1/2 MS medium containing 0.4% phytagel agar for 14 days. Wild-type and GFP- α-tubulin tobacco (Gillespie et al., 2002) were cultured in a growth chamber at 25°C with a 16 h light/8 h dark photoperiod for approximately 4 weeks. The first to third fully expanded true leaves were used for the infiltration experiment.

### Plasmid construction and plant transformation

Amplification of DNA fragments was carried out using KOD-Fx DNA polymerase (Toyobo, Japan). As PCR templates, *Arabidopsis* genomic DNA for cloning the *AtMAP65-3* promoter; rice cDNA for coding region sequence of *OsMAP65-3.1*, *OsMAP65-3.2*, *OsMYB3R2*, and *OsMYB3R2-L*; and a rice genomic DNA preparation for full length genes and promoters of *OsMAP65-3.1* and *OsMAP65-3.2* were used. Gateway cloning technology (Thermo Fisher) and a recombinase system (Vazyme, China) were applied in plasmid construction. The primers used in DNA amplification are listed in **Table S1**, and oligonucleotides used for gene editing are listed in **Table S2**. For the gateway system, the PCR products were first cloned into the vector pENTR4 using BP Clonase (Thermo Fisher), and the resulting pENTR plasmids were recombined with the pGWB659 (containing RFP) and pGWB4 (containing GFP) vectors (Nakagawa et al., 2007) with LR Clonase (Thermo Fisher). For the recombinase system, the PCR products and linearized destination vectors were directly recombined by Exnase II (Vazyme). Gene-editing plasmids were constructed from VK005-09 (Beijing Viewsolid Biotech, China), as described by the manufacturer. Plasmids were transformed into *Agrobacterium tumefaciens* strain GV3101 for infiltration into tobacco plant leaves and transformation of *Arabidopsis*, and strain EHA105 was used for rice transformation. The infiltration of tobacco leaves and a use of a cell-division-enabled leaf system in tobacco pavement cells was as described in our previous work (Xu et al., 2020). *Arabidopsis* plants were transformed *via* the floral dip method (Clough and Bent, 1998), and rice transformation was performed as previously described (Li and Li, 2003).

### Quantitative real-time PCR

Total RNA was isolated from rice tissues with a Takara Plant MiniBEST RNA Extraction Kit (Takara, Japan), and cDNA samples were prepared using PrimeScript II reverse transcriptase (Takara, Japan). Triplicate qPCR amplifications were performed with SYBR Green PCR Master Mix (Applied Biosystems, United States) in a 20 μl reaction using an 7500 Real-Time PCR System (Applied Biosystems). The relative quantification of transcripts was calculated from the 2^-△Ct^ values, which were normalized to that of the ubiquitin gene. Primers used in this experiment are listed in **Table S3**.

### Promoter : GUS analysis

Tissues collected from p*OsMAP65-3s*::*GUS* transformants were stained with a solution containing X-Gluc at 37°C in the dark for 10 h. Chlorophyll was removed by ethanol for observation.

### Fluorescent microscopy

For the immunolocalization of proteins, meristematic cells derived from root tips were fixed and used for indirect immunofluorescence staining according to previously described protocols (Lee and Liu, 2000). The primary antibodies used in this study were rabbit anti-GFP (Invitrogen, USA) and monoclonal anti-alpha-tubulin (DM1A) (Abcam). Secondary antibodies were fluorescein isothiocyanate (FITC)-conjugated donkey anti-mouse IgG, FITC-conjugated donkey anti-rabbit IgG, and Texas-red-conjugated donkey anti-mouse IgG (Invitrogen). Stained cells were observed under an Eclipse Ni-U microscope equipped with MRD71670 and MRD71970 CFI Plan Apochromat Lambda D objectives (Nikon), and images were acquired using a Panda sCMOS camera (PCO Imaging) and the Nikon software package. Leaf samples in the cell-division-enabled leaf system were observed under an Axio Observer inverted microscope equipped with the LSM880 laser scanning confocal module with standard settings for GFP and TaqRFP (Carl Zeiss). Images were acquired with the ZEN software (Zeiss) and processed in IMAGEJ.

### Yeast one-hybrid and two-hybrid assays

For yeast one-hybrid analysis, the full *OsMAP65-3.1* promoter (from 739-bp upstream of the transcriptional start site) and the full *OsMAP65-3.2* promoter (from 374-bp upstream of the transcriptional start site) were amplified and inserted into the pLacZi2µ vector (Takara) to direct the expression of the LacZ reporter. The *OsMYB3R2* or *OsMYB3R2-L* cDNA sequence was cloned into the pB42AD vector (Takara). The constructed pLacZi2µ plasmids inserted by promoters were co-transformed with the pB42AD vectors containing a transcription factor or control (empty pB42AD) vectors into the yeast strain EGY48 using the PEG/LiAc method. Transformed yeast cells were cultured on -Ura/-Trp plates, and then transferred to -Ura/-Trp plates containing 2% (w/v) Gal and 1% (w/v) raffinose to check for possible interactions between OsMYB3R2/OsMYB3R2-L factors and the promoters of *OsMAP65-3* genes.

A two-hybrid system (Clontech) was used in the dimerization assay of OsMAP65-3 proteins. Full-length *OsMAP65-3.1* or *OsMAP65-3.2* CDS were inserted into both bait vector pGBKT7 and prey vector pGADT7 (Clontech). Recombinant plasmids were co-transformed into yeast strain AH109, after which, the transformed yeast strains were grown on -Leu/-Trp plates before being transferred to -Leu/-Trp/-His/-Ade plates to confirm dimerization. pGBKT7-53 and pGADT7-T were used as positive controls. Primers used in the yeast one-hybrid and two-hybrid experiments are listed in **Table S1**.

### Chromatin immunoprecipitation - qPCR assay

Root tips from OsMYB3R2-eGFP and OsMYB3R2-L-eGFP transformants were collected and cross-linked. ChIP assays were performed using a monoclonal anti-eGFP antibody (Sigma-Aldrich) and the ChIP Assay Kit (P2078, Beyotime, Shanghai, China) following the manufacturer’s instructions. Monoclonal anti-eGFP antibody (Sigma-Aldrich) was used. Approximately 10% of sonicated, but non-immunoprecipitated, chromatin was used as an input DNA control. Three replicates of each sample were included. Both ChIP products and input DNA were analyzed by qPCR using region-specific primers (**Table S3**).

## Results

### Rice contains two homologues of MAP65-3

All eleven putative rice MAP65 proteins and nine *Arabidopsis* MAP65 proteins were used for phylogenetic analysis. There were three rice proteins in the MAP65-3/MAP65-4/MAP65-9 clade. To identify MAP65-3 homologues in rice, we further detected the homology relationships among all six proteins in the MAP65-3/MAP65-4/MAP65-9 clade (**Figure S1**). Os01g0685900 and Os05g0552900 had close homology to AtMAP65-3, and the identities between them and AtMAP65-3 were 55% and 53%, respectively, at the amino acid level. MAP65 family members contained conserved N-terminal halves and variable C-terminal halves (including MTB1 and the C-terminal-most MTB2 domains, which exhibited the most divergence) (Smertenko et al., 2008; Ho et al., 2012). Os01g0685900 and Os05g0552900 had higher identity to AtMAP65-3 at the N-terminal and C1 domains, at over 60%. Even in the C2 domain, the identities were 41.70% and 34.55% (**Table S4**). Therefore, *Os01g0685900* and *Os05g0552900* were identified as two hypothetical MAP65-3 homologue genes in rice, and they were named *OsMAP65-3.1* and *OsMAP65-3.2* in this study.

**Figure 1:**
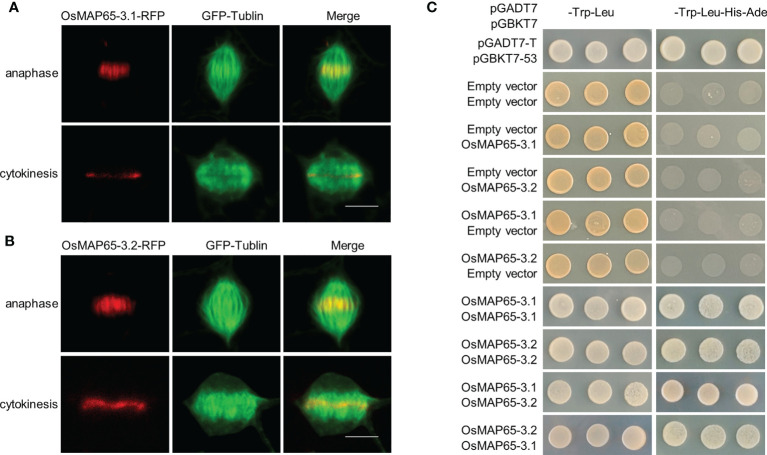
Identification of rice MAP65-3 homologs. **(A, B)** The localization patterns of OsMAP65-3.1 **(A)** and OsMAP65-3.2 **(B)** detected by cell-division-enabled leaf system. OsMAP65 proteins are fused with TagRFP (red), and TUA6 fused GFP are used to label MTs (green). Bars = 10 um. **(C)** Yeast-Two-Hybrid Assay of dimerization of OsMAP65-3.1 and OsMAP65-3.2. The left panel indicate the co-transfomed yeast cells spotted onto nonselective medium lacking Leu and Trp, and the right panel indicate those on selective medium lacking Leu, Trp, His, and Ura.

Because AtMAP65-3 plays primary roles in phragmoplast integrity, the locations of OsMAP65-3.1 and OsMAP65-3.2 in mitosis were rapidly detected using a cell division-enabled leaf system which is established by transient ectopic expression of D-type cyclin inducing mitosis in differentiated cells (tobacco pavement cells) (Xu et al., 2020). OsMAP65-3.1 and OsMAP65-3.2 had similar location patterns in the re-entering cell division: they first appeared at the middle segments of the central spindle MTs at late anaphase, later becoming concentrated at the phragmoplast midzone in telophase, and remained associated with the phragmoplast midline where antiparallel MTs are present in late cytokinesis (**Figures 1A, B**). The location patterns of the two rice MAP65-3s were exactly as that for AtMAP65-3, which was observed by immunofluorescent localization in *Arabidopsis* root tip cells with anti-MAP65-3 antibodies (Ho et al., 2011b). The dimerization of AtMAP65-3 is essential for phragmoplast MT bundling (Smertenko et al., 2008; Ho et al., 2012). The dimerization of OsMAP65-3.1 and OsMAP65-3.2 were detected using yeast two-hybrid analysis. Both OsMAP65-3.1 and OsMAP65-3.2 had the capacity to interact with themselves to form homodimers (**Figure 1C**). In addition, OsMAP65-3.1 also bound to OsMAP65-3.2 to compose a heterodimer (**Figure 1C**). These results suggest that rice contains two MAP65-3 homologues that are likely to have redundant functions in phragmoplast MT dynamics during cytokinesis.

### Both OsMAP65-3 proteins recover the *atmap65-3* phenotype

**Figure 2:**
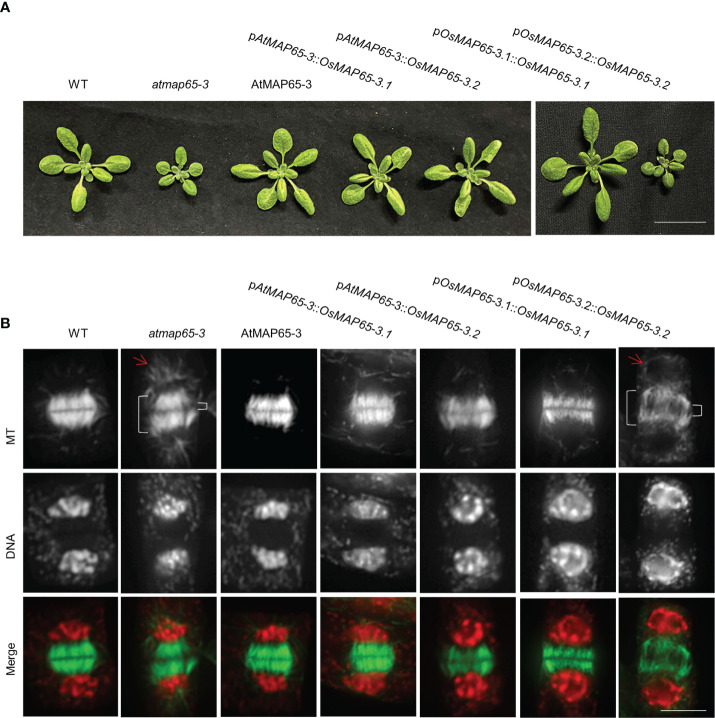
The effects of ectopic expression of *OsMAP65-3.1* and *OsMAP65-3.2* on suppression of the *atmap65-3* phenotypes. **(A)** Growth phenotypes of 21-d-old seedlings and **(B)** developing phragmoplasts MTs of 14-d-old seedlings of the control wild-type, *atmap65-3*, and transformants expressing the *AtMAP65-3*, *OsMAP65-3.1* and *OsMAP65-3.2* proteins with *AtMAP65-3* promoter or their own promoters in *atmap65-3* background. The merged images have MTs in green and DNA in red. The bracket on the left represents the phragmoplast length and that on the right for the gap width, and arrows represents eminent MTs radiating from the two reforming daughter nuclei. Bar = 5 cm **(A)** and 5 um **(B)**.

To further confirm whether OsMAP65-3 proteins function in cytokinesis, *OsMAP65-3.1* and *OsMAP65-3.2* were expressed in the *atmap65-3* mutant (SALK_022166) using the promoter of *AtMAP65-3* to ensuring accurate spatial and temporal expression during *Arabidopsis* cytokinesis. The *atmap65-3* mutant exhibited growth defects (**Figure 2A**), an disorganized phragmoplast MT array (a wider gap in the phragmoplast midzone), and inordinate MTs radiating from the daughter nuclei (**Figure 2B**), which were consistent with published studies (Ho et al., 2011a; Ho et al., 2012). Both OsMAP65-3.1 and OsMAP65-3.2 restored the disordered phragmoplast MT arrays in *atmap65-3* to the normal organization patterns seen in the wild-type cells as effectively as AtMAP65-3 (**Figure 2B**). The phragmoplasts MT bundles were tightly packed and organized in the restored cells. In addition, the eminent MTs that emanated from the surface of the reforming daughter nuclei also disappeared after the expression of *OsMAP65-3.1*, *OsMAP65-3.2*, or *AtMAP65-3* (**Figure 2B**). Finally, the growth defects in *atmap65-3* were resolved by the expression of both rice MAP65-3 proteins (**Figure 2A**). These results suggest that both OsMAP65-3 proteins have similar functions to AtMAP65-3 and play substantial roles in the organization of phragmoplast MTs for cytokinesis and can compensate for a lack of *AtMAP65-3* in *Arabidopsis*.

Subsequently, *OsMAP65-3.1* and *OsMAP65-3.2* whose expression was driven by their native promoters were transformed respectively into *atmap65-3* to test whether these promoters are effective in cytokinesis. Only the p*OsMAP65-3.1*::*OsMAP65-3.1* construct was able to restore seedling growth, while p*OsMAP65-3.2*::*OsMAP65-3.2* had no effect (**Figure 2A**). *OsMAP65-3.1* expression driven by its native promoter restored phragmoplast MT arrays to the normal organization patterns seen in the control cells, and their phragmoplast gap sizes were comparable to those of the wild-type cells (**Figure 2B**); while p*OsMAP65-3.2*::*OsMAP65-3.2*-introduced plants showed loosely packaged and disorganized phragmoplasts, similar to *atmap65-3* (**Figure 2B**). These results indicate that the *OsMAP65-3.1* promoter has a similar effect to that of *AtMAP65-3*, but *OsMAP65-3.2* promoter is unable to drive its expression in *Arabidopsis*, leading to its inaction in cytokinesis. This conclusion was further confirmed by immunofluorescence with an anti-GFP antibody to detect the expression and location of OsMAP65-3.1 fused with GFP. OsMAP65-3.1 fused with GFP was expressed in anaphase, telophase, and cytokinesis, and it was mainly located in the phragmoplast midzone. However, when using the *OsMAP65-3.2* promoter, no GFP immune signal was observed at any mitosis phase in root cells (**Figure S2**). Therefore, the functional differences between *OsMAP65-3.1* and *OsMAP65-3.2* with regard to phragmoplast organization in the *atmap65-3* mutant were due to differences in their expression patterns and had no connection with their amino acid sequence diversity.

### Different expression patterns of *OsMAP65-3* genes in rice

**Figure 3:**
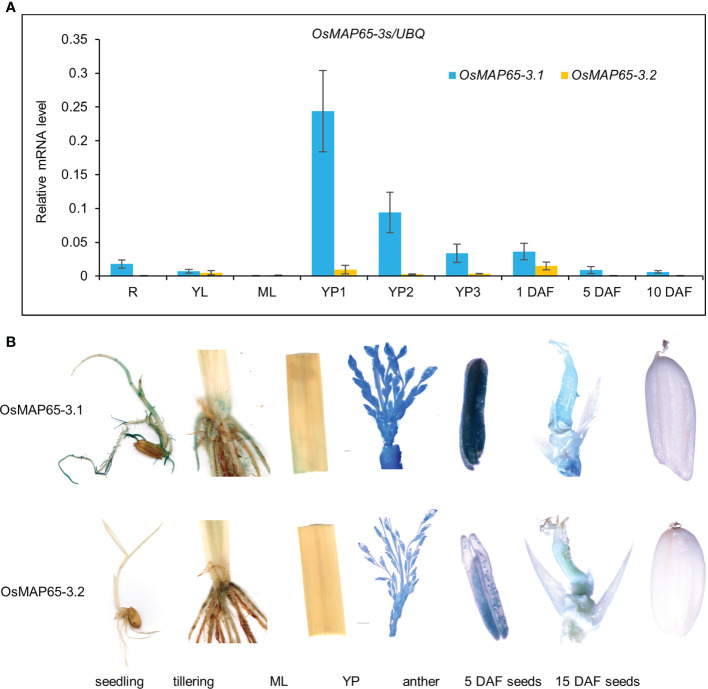
Expression patterns of *OsMAP65-3.1* and *OsMAP65-3.2* in rice. **(A)** Transcriptional levels of *OsMAP65-3.1* and *OsMAP65-3.2* in various tissues, including the roots (R), young leaves (YL), mature leaves (ML), YP1–3 (young inflorescences < 2 cm, 2 – 5 cm, > 5 cm), 1 DAF spikelets (1day after fertilization), 5 DAF and 10 DAF seeds. The data presented are the means ± SDs of three biological replicates. **(B)** GUS staining of transgenic rice plants harboring p*OsMAP65-3s*::*GUS*. The detected tissues included seedlings, tillering sites, mature leaves, young panicles, anthers, 5 DAF and 10 DAF seeds.

The expression patterns of *OsMAP65-3.1* and *OsMAP65-3.2* were analyzed in rice. qPCR was used to analyze the transcriptional levels of the genes in several tissues, including 2-week-seedling roots and leaves, heading-stage flag leaves, young inflorescences (<2 cm, 2–5 cm, >5 cm), and spikelets (1 day after fertilization [DAF]), seeds (5 and 10 DAF). *OsMAP65-3.1* was highly expressed in all tissues containing mitotic cells, such as roots, young inflorescences, 1-DAF spikelets, and 5-DAF seeds. The expression level was the highest in young inflorescences shorter than 2 cm, 15-fold higher than in the roots. However, almost no *OsMAP65-3.1* transcripts were detected in the mature leaves, where cell division had largely ceased. The transcriptional level of *OsMAP65-3.2* was extremely low in all tissues and much less than that of *OsMAP65-3.1* (**Figure 3A**). The *OsMAP65-3* genes expression patterns were further confirmed by β-glucuronidase (GUS) assay of p*OsMAP65-3s*::*GUS*-transformed rice plants, and the results were consistent with those of the qPCR analyses. *GUS* expression driven by the *OsMAP65-3.1* promoter resulted in strong staining in the roots, tillering-formation sites, young inflorescences, anthers, pollen, and 5-DAF seeds, while the staining for GUS expressed with the *OsMAP65-3.2* promoter was much paler than that driven by the *OsMAP65-3.1* promoter in every tissue and produced almost no signal in the roots, seedlings, leaves, and 15-DAF seeds (**Figure 3B**).

**Figure 4:**
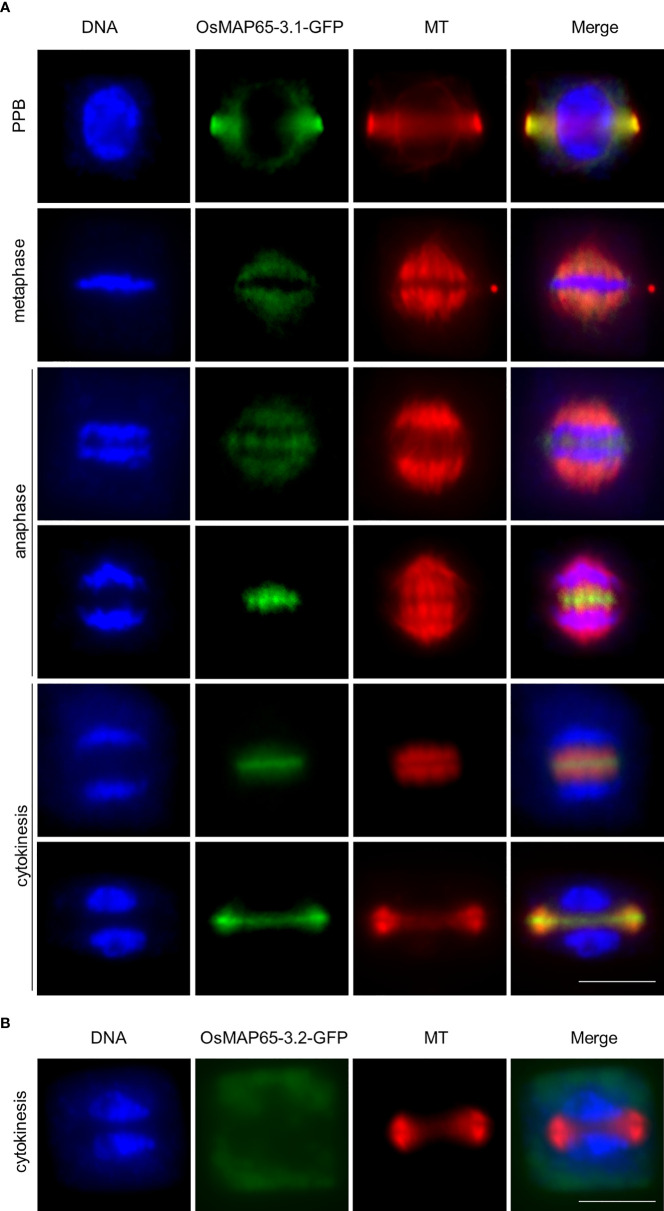
Localization of OsMAP65-3.1 and OsMAP65-3.2 in rice root meristematic cells. **(A)** Immunofluorescent localization reveals that OsMAP65-3.1-GFP binds to MTs (PPB, spindles and phragmoplast) in all mitotic phases including prophase, metaphase, anaphase, telophase and cytokinesis. **(B)** No OsMAP65-3.2-GFP signal is observed at phragmoplast MTs in cytokinesis. Bar = 10 um **(A, B)**.

To examine the location of the OsMAP65-3 proteins during rice mitosis, rice wild-type plants were transformed, respectively, with their genes which were fused with GFP and were expressed using their native promoters. Root apical meristematic cells were collected from transgenic plants for immunofluorescence with a GFP-specific antibody. As a MT-associated protein, OsMAP65-3.1 fused with GFP was observed during the entire process of cell division, including preprophase microtubule band (PPB), spindles in metaphase, spindles and phragmoplast in anaphase, and finally was mainly concentrated at the phragmoplast midzone (**Figure 4A**). Whereas, in the roots of p*OsMAP65-3.2*::*OsMAP65-3.2-GFP* plants, no obvious GFP immunofluorescence signal was evident at any of the mitosis phases, including cytokinesis, in which MAP65-3 proteins are known to function (**Figure 4B** and **Figure S3**). The locational differences between the OsMAP65-3 proteins in rice are similar to their locations in *atmap65-3*, and this discrepancy is possibly resulted from differences in their promoter activity. However, *OsMAP65-3.1* was localized along all MT arrays during cell division in rice, while *AtMAP65-3.1* was mainly localized at the phragmoplast midline since later anaphase; in *atmap65-3*, location of OsMAP65-3.1 was truly the same in every particular as AtMAP65-3, indicating a difference in regulatory mechanisms between monocots and dicots. Overall, the expression patterns and locations suggest that *OsMAP65-3.1*, rather than *OsMAP65-3.2*, plays a primary role in mitosis of rice root meristic cells.

### *OsMAP65-3.1* plays essential roles in rice development

**Figure 5:**
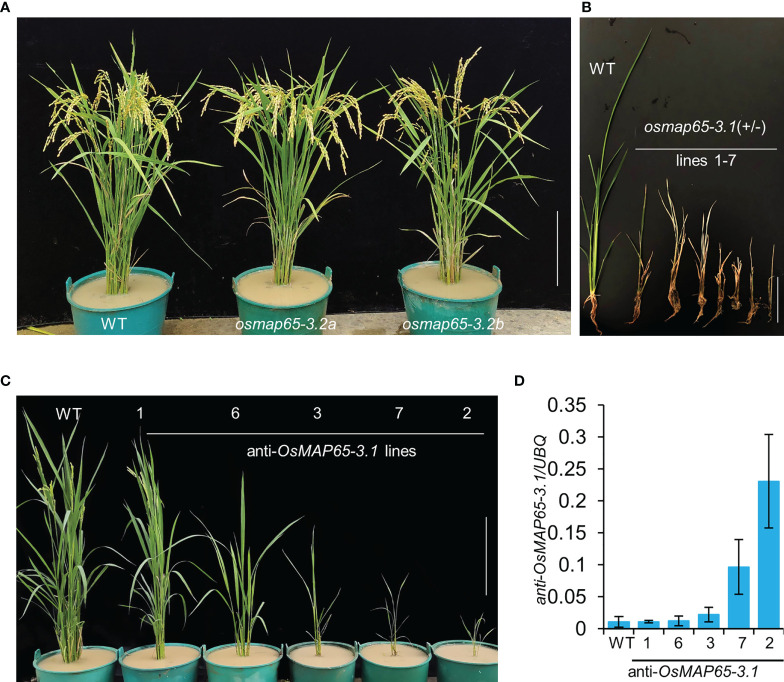
Developmental phenotypes of *osmap65-3.1* and *osmap65-3.2* defect plants. **(A)** Phenotypes of the wild-type and *osmap65-3.2* mutants at the filling stages. **(B)** Lethal phenotype of *osmap65-3.1(+/-)* at the seedling stage. **(C)** Suppressed growth of anti-*OsMAP65-3.1* plants; **(D)** Transcriptional levels of anti-sense *OsMAP65-3.1*. The roots at the seedling stage were used for qRT-PCR, and the *UBQ* gene was used as a control. Mean and SD values were obtained from three biological replicates. Bar = 20 cm **(A, C)**, 5 cm **(B)**.

To further explore the functions of the *OsMAP65-3* genes as well as their divergent roles in the development of rice plants, we knocked out *OsMAP65-3.1* or *OsMAP65-3.2* using the CRISPR/Cas9 system. *OsMAP65-3.2* could be efficiently edited, leading to null alleles, and several plants with two null alleles were even obtained at T_0_ generation, The *osmap65-3.2* plants free of the Cas9 and gRNA construct were isolated from the T_1_ generation. The phenotypes of *osmap65-3.2a* (1-bp-insertion in the first exon) and *osmap65-3.2b* (4-bp-deletion in the first exon) were normal (**Figure S4B** and **Figure 5A**), and there were no significant differences in grain length, grain width, pollen fertility, and most agronomic traits between the *osmap65-3.2* lines and wild-type (**Figure S5** and **Table S5**).

Unexpectedly, the editing of *OsMAP65-3.1* by CRISPR/Cas9 system was hard to achieve. We constructed four gRNA vectors to target different sites in the *OsMAP65-3.1* gene, and only seven heterozygotes containing one null allele were generated in more than 300 transformed plants (**Figure S4A**). None of *osmap65-3.1* homozygotes could be obtained because the heterozygote plants perished at the seedling stage (**Figure 5B**). These results suggest that *OsMAP65-3.1* truly is important in rice, as even half the normal quantity of functional OsMAP65-3.1 resulted in serious disruption to growth and development. We subsequently employed an antisense-RNA strategy to generate *OsMAP65-3.1*-knock-down plants. Antisense of a partial *OsMAP65-3.*1 CDS fragment guided by two tandem 35S promoters was expressed in wild-type plants. In total, 32 antisense-*OsMAP65-3.*1 plants were obtained; nine of them died within a short period of time, and the rest were transplanted to a paddy field and their growth was repressed at different levels (**Figure 5C**). The growth inhibition was proportionate to expression level of the antisense RNA (**Figure 5D**). Line 7 and Line 2, in which antisense RNA was highly expressed, died later during cultivation in the paddy field. Therefore, normal levels of *OsMAP65-3.1* transcripts are necessary for rice development, while *OsMAP65-3.2* appears non-functional or redundant.

### OsMAP65-3.1 is necessary for cytokinesis

**Figure 6:**
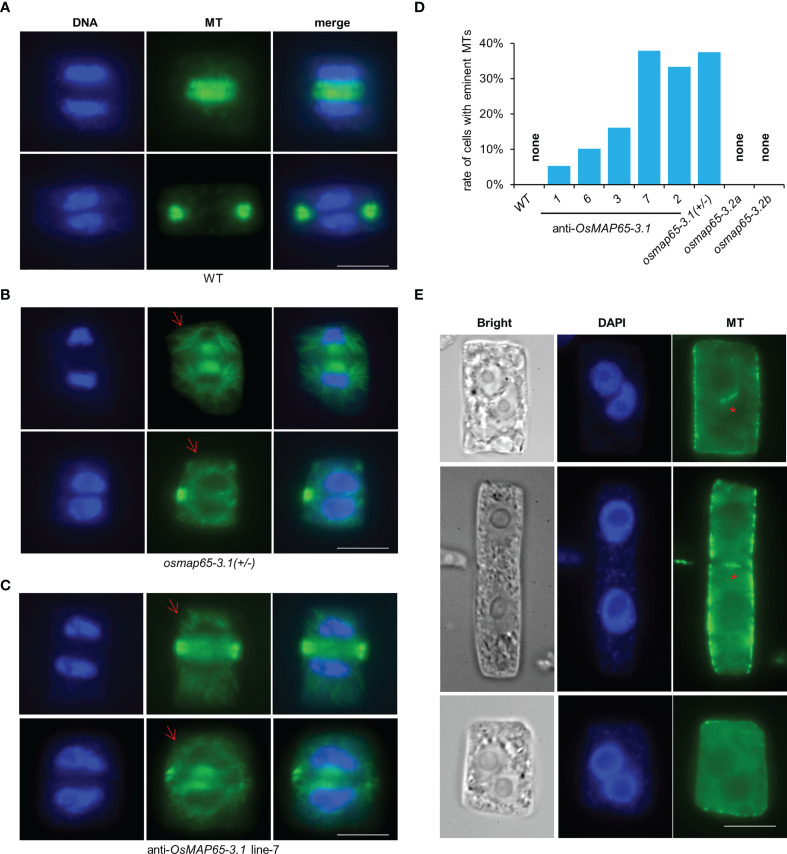
The developing phragmoplasts and cytokinesis in root apical meristematic cells of *osmpa65-3* defect plants. **(A)** Wild-type cells in cytokinesis. **(B, C)** Cells of *osmap65-3.1(+/-)* **(B)** and anti-sense *OsMAP65-3.1* line-7 **(C)** in cytokinesis. Eminent MTs emanated from the surface of the reforming daughter nuclei (the red arrows) were frequently observed. **(D)** The percentage of cells with eminent MTs radiating from daughter nuclei in wild-type, anti-sense *OsMAP65-3.1* lines, *osmap65-3.1(+/-)* and *osmap65-3.2*. **(E)** The cytokinesis failures in *osmpa65-3.1* defect plants. The new cell plates cannot be formed at all or be partially generated (the red stars) in these cells. Bar = 10 um **(A-C, E)**.

MAP65-3 plays essential roles in plant cytokinesis, and OsMAP65-3.1 binds to MTs in mitosis, suggesting OsMAP65-3.1 is a key factor in cell division. Root apical meristematic cells were collected from the *osmap65-3.1* heterozygote and *OsMAP65-3.1*-knock-down plants when they were still alive. A tubulin antibody and immunofluorescence labelling were used to reveal the MT dynamics in mitotic cells. The PPB and spindles exhibited no obvious differences compared to wild-type (**Figure S6**), while phragmoplast organization and cell plate expansion were impacted in some cells. Eminent MTs emanating from the surface of reforming daughter nuclei were frequently observed in the roots of the *OsMAP65-3.1-*defect plants (**Figure 6B**), while no obvious MTs were seen around the daughter nuclei in wild-type plants (**Figure 6A**). In fact, 38.1% of *osmap65-3.1* heterozygote root cells contained abundant MTs radiating from the surface of the nuclear envelope (**Figure 6D**). The eminent MTs were also observed in the *OsMAP65-3.1*-knock-down plants (**Figure 6C**), and their occurrence rates were positively associated with the degree of OsMAP65-3.1 disturbance; the rate was about 5.2% in Line 1, which developed relatively normally, but over 30% in Line 7 and Line 2, whose growth were severely inhibited (**Figure 6D**). Published studies show that the loss of *AtMAP65-3* leads to a wider gap in the phragmoplast midzone compared with that in wild-type cells, as well as eminent MTs radiating from the daughter nuclei (Müller et al., 2004; Ho et al., 2011a). However, the phragmoplast gap in the *osmap65-3.1* heterozygotes and *OsMAP65-3.1*-knock-down plants (even in cells with eminent MTs) were comparable to those of the wild-type control cells (**Figures 6B, C**). Similar to the *atmap65-3* mutants, *OsMAP65-3.1*-defect plants displayed obvious cytokinesis failures. A percentage of the root apical meristematic cells without phragmoplast signals contained two nuclei, indicating that nuclear division had been completed, but the new cell plates could not form or were partially generated (**Figure 6E**).

We also examined mitotic processes in *osmap65-3.2* root cells with immunofluorescence microscopy. The MTs of the PPB, spindles, and phragmoplast in *osmap65-3.2* showed no significant deviation from those in WT (**Figure S7**). The results suggest that OsMAP65-3.2 has no effects on mitosis of root cells and little influence on rice development. Taken together, the evidence we gathered shows that OsMAP65-3.1 plays important roles in cytokinesis, which is essential for rice development.

### Binding of OsMYB3R transcription factors to OsMAP65-3 promoters determines their expression patterns

To clarify the molecular mechanism of the obvious expressional differences between *OsMAP65-3.1* and *OsMAP65-3.2*, the promoters of *OsMAP65-3* genes and *AtMAP65-3* were intricately analyzed to screen for cis-acting elements (**Table S6**). A mitosis-specific activator (MSA) element was discovered within the promoter regions of *OsMAP65-3.1* and *AtMAP65-3*, but not the *OsMAP65-3.2* promoter. The MSA elements in the *OsMAP65-3.1* and *AtMAP65-3* promoters were very near to the transcription initiation site, which is located at positions −234 to −229 and −99 to −94, respectively, suggesting that the MSA element plays key roles in activating the expression of *OsMAP65-3.1* and *AtMAP65-3* (**Table S6**).

**Figure 7:**
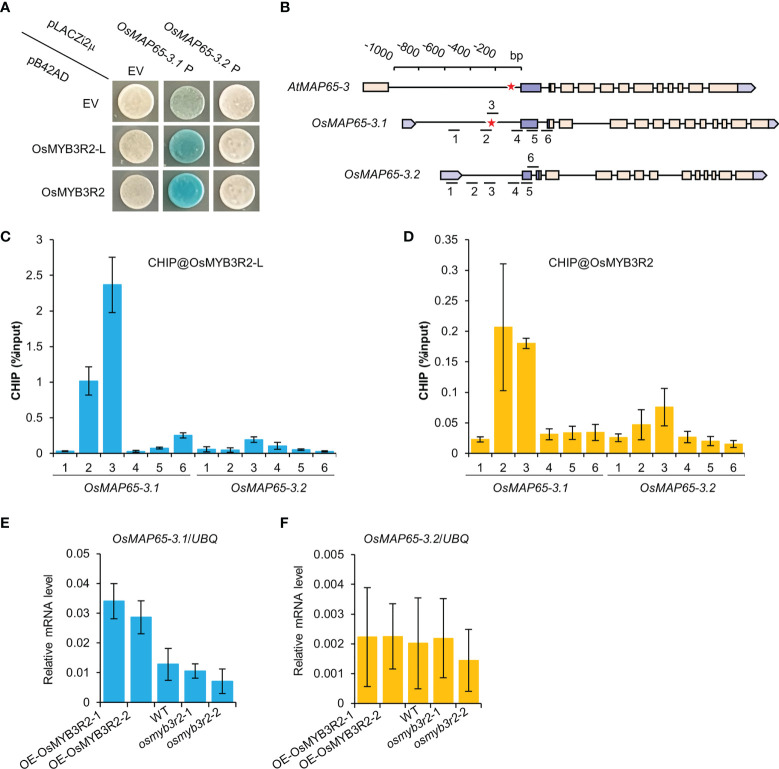
The interactions of OsMYB3R2 transcription factors with the *OsMAP65-3* genes promoters. **(A)** Yeast-one-hybrid assay showing that OsMYB3R2 and OsMYB3R2-L can bind to the *OsMAP65-3.1* promoter but not *OsMAP65-3.2* promoter. **(B)** The position of MSA element and the primers used for ChIP in the promoters of *AtMAP65-3*, *OsMAP65-3.1* and *OsMAP65-3.2*. The red asterisk represents the position of the MSA element. **(C, D)** ChIP-qPCR results showing that OsMYB3R2-L **(C)** and OsMYB3R2 **(D)** bind to *OsMAP65-3.1* promoter rather than *OsMAP65-3.2* promoter, and OsMYB3R2-L and OsMYB3R2 were enriched at the region covered by ChIP-qPCR primer 2 and 3. Data are shown as means ± SD (n = 3). **(E, F)** The transcription level of *OsMAP65-3.1* **(E)** and *OsMAP65-3.2* **(F)** in OsMYB3R2 overexpression and knockout plants.

In plants, MYB3R transcription factors bind to MSA elements and regulate the expression of the late-cell-cycle genes (in G2 and M phases) (Haga et al., 2011; Magyar et al., 2016). In the rice genome, there are four MYB3R genes: *Os12g0238000* (*OsMYB3R1*), *Os01g0229000* (*OsMYB3R1-L*), *Os01g0841500* (*OsMYB3R2*), and *Os05g0459000* (*OsMYB3R2-L*) (**Figure S8**). It has been demonstrated that OsMYB3R2 functions as a positive regulator of the G2/M phase of the cell cycle in rice (Ma et al., 2009). Yeast-one hybrid analysis was carried out to identify the binding of OsMYB3R2 and OsMYB3R2-L to the promoters of the *OsMAP65-3* genes. Both OsMYB3R2 and OsMYB3R2-L were found to bind to the *OsMAP65-3.1* promoter in yeast, but not to the *OsMAP65-3.2* promoter (**Figure 7A**). We subsequently performed ChIP-qPCR analysis to test if OsMYB3R2 and OsMYB3R2-L bind to *OsMAP65-3* genes promoters *in vivo*. ChIP-qPCR results demonstrated that the two OsMYB3R proteins were significantly enriched at the target region, i.e., the location of the closest MSA element in the *OsMAP65-3.1* promoter (**Figures 7B-D**), while neither OsMYB3R2 nor OsMYB3R2-L showed significant enrichment at the *OsMAP65-3.2* promoter, which contains no MSA element (**Figures 7C, D**). Finally, we examined the abundance of *OsMAP65-3* genes mRNAs at the root tips of *OsMYB3R2*-overexpression and -knockout plants which were confirmed by qPCR and genotyping of *OsMYB3R2* (**Figure S9**). In the overexpression plants, *OsMAP65-3.1* was significantly up-regulated, while the expression of *OsMAP65-3.2* did not change significantly (**Figure 7E**), further supporting the conclusion that OsMYB3R2 is a transactivator of *OsMAP65-3.1* (**Figure 7F**). However, the expression levels of *OsMAP65-3* genes were not significantly influenced in either *osmyb3r2* or *osmyb3r2-l* (**Figures 7E, F** and **Figure S10**), which may be due to the functional redundancy of the OsMYB3R transcription factors in the transactivation of *OsMAP65-3.1*.

## Discussion

### Functional differences between OsMAP65-3.1 and OsMAP65-3.2 in rice development result from their different expression patterns

The MAP65/Ase1/PRC1 family of MAP proteins have evolutionarily conserved functions in MT organization (Walczak and Shaw, 2010), and both monocot and dicot plants contain a large number of proteins of this family (Smertenko et al., 2008; Guo et al., 2009). MAP65-3 plays a dominant role in phragmoplast MT organization, which is essential for cytokinesis, and OsMAP65-3.1 and OsMAP65-3.2 are MAP65-3 homologues found in rice based on similarity of the amino acid sequences (**Figure S1**); however, their roles in rice development are quite different. The *osmap65-3.2* mutant plants were found to have no significantly abnormal phenotype, while both the homozygote and most heterozygotes of *osmap65-3.1* were nonviable at the seedling stage (**Figure 5**, **Figure S5** and **Table S5**).

MAP65 family proteins have a relatively conserved N-terminal for dimerization and a variable C-terminal for MT binding (Smertenko et al., 2008). OsMAP65-3.1 and OsMAP65-3.2 are highly homologous, with 78% identity at the N-terminal and 82% identity at the C-terminal MTB1 domain. Even at the MTB2 domain, which usually has the most divergence, the homology between the two OsMAP65 homologues was as high as 61%. Moreover, both OsMAP65-3.1 and OsMAP65-3.2 were able to form a homodimer and interact with each other to form a heterodimer (**Figure 1C**). Both OsMAP65-3s could bind to phragmoplast MTs, were enriched at the middle zone in telophase (**Figures 1A, B**), and were able to recover the cytokinesis-failure phenotype of *atmap65-3* (**Figure 2**). These results revealed that OsMAP65-3.1 and OsMAP65-3.2 proteins have no functional differences and both function in cytokinesis. However, a series of evidences demonstrated that only *OsMAP65-3.1* promoter is active in cytokinesis: a) only the p*OsMAP65-3.1*::*OsMAP65-3.1* recovered the phenotypes of *atmap65-3*, while p*OsMAP65-3.2*::*OsMAP65-3.2* was nonfunctional (**Figure 2**); b) the significant differences did exist in expression patterns of *OsMAP65-3* genes. The transcriptional level of *OsMAP65-3.2* was extremely low in all tissues and much less than that of *OsMAP65-3.1* (**Figure 3**); c) OsMAP65-3.1-GFP was observed in root apical meristematic cells of both *Arabidopsis* and rice using immunofluorescence method, while no obvious OsMAP65-3.2-GFP signal was evident at any of the mitosis phases (**Figure 4** and **Figure S2**). All evidences pointed to the conclusion that the functional differences between *OsMAP65-3.1* and *OsMAP65-3.2* in rice result from divergences in their gene expression, rather than their encoded proteins.

Interestingly, compared to none expression in rice root, OsMAP65-3.2 had different localization patterns in tobacco leaf system. Using CDELS, we observed both OsMAP65-3.1-TaqRFP and OsMAP65-3.2-TaqRFP at the middle segments of the central spindle MTs at late anaphase and later becoming concentrated at the phragmoplast midzone in telophase (**Figure 1**). Since the CDELS is established by transient ectopic expression of D-type cyclin inducing mitosis in differentiated cells. We hypothesize that CDELS is a re-entering cell division of differentiated cells which might be different from normal mitosis in root apical meristematic cells; and the transient ectopic expression of *OsMAP65-3.2* promoter is possibly activated in tobacco leaf cells before re-entering mitosis, then OsMAP65-3.2 is expressed and observed at mitotic microtubule arrays.

### Role of transcriptional factors in regulation of *MAP65-3* expression during cytokinesis

In animal and plant cells, the expression of early-cell-cycle genes is thought to be induced by the activator-type E2F family of transcription factors (Berckmans and De Veylder, 2009; Chen et al., 2009), but it is becoming apparent that E2Fs are not required to drive cell proliferation (Chen et al., 2009; Wang et al., 2014). In plants, late-cell-cycle genes are regulated by MYB3R transcription factors (Ito, 2005). The Arabidopsis *myb3r1 myb3r4* double-mutant showed incomplete cytokinesis during somatic cell division, which was characterized by binucleate guard cells with irregularly shaped stomata, gapped epidermal cell walls, and single-celled embryos with multiple nuclei (Haga et al., 2007). In this double-mutant, a large number of late-cell-cycle genes were down-regulated resulting from the cis-acting MSA element in their promoters where MYB3R factors bind (Haga et al., 2011). The evidence further pointed to the role of MYB3R transcription factors in positively regulating cytokinesis, mainly through the transcriptional activation of the *KNOLLE* gene containing MSA motifs in promoter (Haga et al., 2007). *AtKNOLLE* encodes a cytokinesis-specific and plant-specific vesicle fusion SNARE protein and functions in cell plate formation (Enami et al., 2009). The cytokinesis defects in *myb3r1 myb3r4* were effectively, but not completely, recovered by the introducing of *KNOLLE*, whose expression is directed by a heterologous promoter and the activity of which is independent of MSA elements (Haga et al., 2007). Hence, MYB3R transcription factors positively regulate other key factors in cytokinesis besides KNOLLE, and these might include MAP65-3.

In this study, the *atmap65-3* complementation test indicated that both the *AtMAP65-3* and *OsMAP65-3.1* promoters were activated in cytokinesis, but the *OsMAP65-3.2* promoter was not. We further found that *AtMAP65* and *OsMAP65-3.1* contain MSA motifs in their promoter regions, while *OsMAP65-3.2* had no MSA (**Table S6**). Subsequently, two rice MYB3R homologues, OsMYB3R2 and OsMYB3R2L, were demonstrated to directly bind to the *OsMAP65-3.1* promoter, but not the *OsMAP65-3.2* promoter, and activated the *OsMAP65-3.1* promoter in both vivo and vitro, while having no significant effects on the *OsMAP65-3.2* promoter (**Figure 7**). Therefore, the expression of *MAP65-3* in the cell cycle might be activated by MYB3R transcription factors, and the functional differences between *OsMAP65-3.1* and *OsMAP65-3.2* probably relate to whether or not they have an MYB3R-binding site (MSA element) in their promoter. However, it is not clear whether other transcription factors are also responsible for the differential expression patterns of the *OsMAP65-3* genes. Interestingly, we found a TTTCCCCC motif in the *OsMAP65-3.2* promoter, which might be an E2F binding site (Vandepoele et al., 2005), that was not found in the *OsMAP65-3.1* or *AtMAP65-3* promoters. E2F transcriptional factors comprise repressor- and activator-types, and the E2FC associations with MYB3R3 and RETINOBLATOMA RELATED (RBR) form repressor-type DREAM-like complexes involved in repressing the expression of cell cycle genes (Kobayashi et al., 2015; Magyar et al., 2016). Thus, more explorations are needed into the effects of OsE2Fs or DREAM on the suppression of *OsMAP65-3.2* expression.

### OsMAP65-3.1 may serve functions in rice that are not shared by AtMAP65-3 in *Arabidopsis*

The *atmap65-3* null mutant shows severe cytokinesis defects, that are linked to compromised seedling growth (Müller et al., 2004; Ho et al., 2012). *OsMAP65-3.1* suppressed the phenotypes caused by the loss of AtMAP65-3 in *Arabidopsis*, suggesting that OsMAP65-3.1 and AtMAP65-3 have similar functions in cytokinesis. The *atmap65-3* mutant was still able to undergo vegetative growth and sexual reproduction; whereas no viable *osmap65-3.1* homozygotes were obtained under normal growth conditions (**Figure 6A**), suggesting that OsMAP65-3.1 plays a much more decisive role in rice cytokinesis than AtMAP65-3 in *Arabidopsis*. Alternatively, OsMAP65-3.1 may possess functions beyond that in the phragmoplast midzone. This notion is supported by its localization pattern when compared to that of AtMAP65-3. OsMAP65-3.1 was highly abundant in the PPB and spindles prior to the formation of the phragmoplast microtubule array. In contrast, AtMAP65-3 is not detected on spindle microtubules (Ho et al., 2011a). It would be interesting to learn whether the loss of OsMAP65-3.1 would lead to defects in spindle assembly in additional the cytokinetic phenotypes documented for AtMAP65-3.

In *Arabidopsis*, several MAP65 members revealed certain degrees of functional redundancy with MAP65-3. Previous studies showed that the double-mutants *map65-1 map65-3* and *map65-2 map65-3* exhibited enhanced phenotypes involving seriously dwarfed seedlings (Sasabe et al., 2011; Lucas and Shaw, 2012), while the *atmap65-4 atmap65-3* double-mutant was synthetically lethal, and even the *atmap65-3*(-/-);*atmap65-4*(+/-) mutant exhibited enhanced growth defects compared to the *map65-1 map65-3* double-mutant (Li et al., 2017). All three AtMAP65 proteins were postulated to share similar functions with MAP65-3. AtMAP65-4, with the highest homology to AtMAP65-3, acts as a more effective complement to non-functional AtMAP65-3 than others, and the introducing of an extra copy of the *AtMAP65-4* gene completely recovers the defective growth caused by the *atmap65-3* null mutation, indicating that the effect of one *AtMAP65-4* copy in cytokinesis is roughly equivalent to half the effects of AtMAP65-3 (Li et al., 2017). Although more MAP65 members are included in rice than *Arabidopsis* (Guo et al., 2009), no other MAP65 proteins effectively compensated for the loss of OsMAP65-3.1 in rice. According to the inactive promoter, OsMAP65-3.2, which has high homology to OsMAP65-3.1 (**Figure S1**), had no effects on cytokinesis; while the effects of other MAP65 members with low homology to OsMAP65-3.1 might be minor, similar to AtMAP65-1 and AtMAP65-2 in *Arabidopsis*.

Hence, in both rice and *Arabidopsis*, a sufficient quantity of MAP65 protein during cytokinesis is necessary for normal growth. The quantity of AtMAP65-3 and OsMAP65-3.1 in wild-type plants, and the two copies of *AtMAP65-4* in *atmap65-3*, are enough for normal growth. AtMAP65-4 combined with AtMAP65-1 and AtMAP65-2 (*atmap65-3*), a combination of AtMAP65-4 and AtMAP65-1 or AtMAP65-2 (*atmap65-3 atmap65-1* or *atmap65-3 atmap65-2* double mutant), and half the amount of AtMAP65-4 combined with AtMAP65-1 and AtMAP65-2 (*atmap65-3(-/-);atmap65-4(+/-)*) are inadequate for normal development, but these plants are able to survive. Combinations of other MAP65 members without AtMAP65-4 and AtMAP65-3 in *Arabidopsis* (*atmap65-3 atmap65-4*) and without OsMAP65-3.1 in rice (*osmap65-3.1*) only provide a small quantity of MAP65 for cytokinesis and are synthetically lethal. Surprisingly, half the amount of MAP65-3 in rice (*osmap65-3.1(+/-)*) failed to meet the minimum requirements for survival, leading to seedling death, while *atmap65-3*, which contains approximately half the amount of MAP65-3, can undergo a full life cycle because of the presence of functional AtMAP65-4. This discrepancy might result from the different culture conditions of rice and *Arabidopsis*; *Arabidopsis* is usually cultured in a greenhouse growth chamber under optimum conditions, while rice is typically cultivated in paddy fields and is subject to uncontrollable external environmental conditions, especially heat stress, and thus may need to recruit more active cell division in response to stresses. Thus, we speculated that more MAP65-3 is need for rice survival than *Arabidopsis* survival, and half the quantity of OsMAP65-3.1 is thus inadequate.

## Data availability statement

The raw data supporting the conclusions of this article will be made available by the authors, without undue reservation.

## Author contributions

JX, HH, BL and XD planned and designed the research. XL, YX, YS, CG, PW, JL, ZJ, LP, DZ, XH, JB and CZ performed experiments and analysed data. XL, JX, HH and BL wrote and revised the manuscript. XL, YX and YS contributed equally. All authors contributed to the article and approved the submitted version.

## Funding

This work was supported by the National Science Foundation of China (Grant Nos. 31960403), Jiangxi Natural Science Foundation (Grant NO. 20181BCD41002), and Jiangxi Natural Science Foundation-Outstanding Youth Science Fund Project (Grant No. 20212ACB215003).

## Conflict of interest

The authors declare that the research was conducted in the absence of any commercial or financial relationships that could be construed as a potential conflict of interest.

## Publisher’s note

All claims expressed in this article are solely those of the authors and do not necessarily represent those of their affiliated organizations, or those of the publisher, the editors and the reviewers. Any product that may be evaluated in this article, or claim that may be made by its manufacturer, is not guaranteed or endorsed by the publisher.

## References

[B1] Ambrose J. C. Shoji T. Kotzer A. M. Pighin J. A. Wasteneys G. O. (2007). The *Arabidopsis CLASP* gene encodes a microtubule-associated protein involved in cell expansion and division. Plant Cell. 19, 2763–2775. doi: 10.1105/tpc.107.053777 17873093 PMC2048705

[B2] Beck M. Komis G. Müller J. Menzel D. Samaj J. (2010). *Arabidopsis* homologs of nucleus- and phragmoplast-localized kinase 2 and 3 and mitogen-activated protein kinase 4 are essential for microtubule organization. Plant Cell. 22, 755–771. doi: 10.1105/tpc.109.071746 20215588 PMC2861451

[B3] Beck M. Komis G. Ziemann A. Menzel D. Šamaj J. (2011). Mitogen-activated protein kinase 4 is involved in the regulation of mitotic and cytokinetic microtubule transitions in *Arabidopsis thaliana* . New Phytol. 189, 1069–1083. doi: 10.1111/j.1469-8137.2010.03565.x 21155826

[B4] Berckmans B. De Veylder L. (2009). Transcriptional control of the cell cycle. Curr. Opin. Plant Biol. 12, 599–605. doi: 10.1016/j.pbi.2009.07.005 19700366

[B5] Bisgrove S. R. Lee Y. R. Liu B. Peters N. T. Kropf D. L. (2008). The microtubule plus-end binding protein EB1 functions in root responses to touch and gravity signals in *Arabidopsis* . Plant Cell. 20, 396–410. doi: 10.1105/tpc.107.056846 18281505 PMC2276450

[B6] Boruc J. Weimer A. K. Stoppin-Mellet V. Mylle E. Kosetsu K. Cedeño C.. (2017). Phosphorylation of MAP65-1 by *Arabidopsis* aurora kinases is required for efficient cell cycle progression. Plant Physiol. 173, 582–599. doi: 10.1104/pp.16.01602 27879390 PMC5210758

[B7] Caillaud M. C. Lecomte P. Jammes F. Quentin M. Pagnotta S. Andrio E.. (2008). MAP65-3 microtubule-associated protein is essential for nematode-induced giant cell ontogenesis in *Arabidopsis* . Plant Cell. 20, 423–437. doi: 10.1105/tpc.107.057422 18263774 PMC2276437

[B8] Chen D. Pacal M. Wenzel P. Knoepfler P. S. Leone G. Bremner R. (2009). Division and apoptosis of E2f-deficient retinal progenitors. Nature 462, 925–929. doi: 10.1038/nature08544 20016601 PMC2813224

[B9] Clough S. J. Bent A. F. (1998). Floral dip: a simplified method for *Agrobacterium*-mediated transformation of *Arabidopsis thaliana* . Plant J. 16, 735–743. doi: 10.1046/j.1365-313x.1998.00343.x 10069079

[B10] Enami K. Ichikawa M. Uemura T. Kutsuna N. Hasezawa S. Nakagawa T.. (2009). Differential expression control and polarized distribution of plasma membrane-resident SYP1 SNAREs in *Arabidopsis thaliana* . Plant Cell Physiol. 50, 280–289. doi: 10.1093/pcp/pcn197 19098073

[B11] Gillespie T. Boevink P. Haupt S. Roberts A. G. Toth R. Valentine T.. (2002). Functional analysis of a DNA-shuffled movement protein reveals that microtubules are dispensable for the cell-to-cell movement of *tobacco mosaic virus* . Plant Cell. 14, 1207–1222. doi: 10.1105/tpc.002303 12084822 PMC150775

[B12] Glotzer M. (2001). Animal cell cytokinesis. Annu. Rev. Cell Dev. Biol. 17, 351–386. doi: 10.1146/annurev.cellbio.17.1.351 11687493

[B13] Guo L. Ho C. M. Kong Z. Lee Y. R. Qian Q. Liu B. (2009). Evaluating the microtubule cytoskeleton and its interacting proteins in monocots by mining the rice genome. Ann. Bot. 103, 387–402. doi: 10.1093/aob/mcn248 19106179 PMC2707338

[B14] Haga N. Kato K. Murase M. Araki S. Kubo M. Demura T.. (2007). R1R2R3-myb proteins positively regulate cytokinesis through activation of *KNOLLE* transcription in *Arabidopsis thaliana* . Development 134, 1101–1110. doi: 10.1242/dev.02801 17287251

[B15] Haga N. Kobayashi K. Suzuki T. Maeo K. Kubo M. Ohtani M.. (2011). Mutations in *MYB3R1* and *MYB3R4* cause pleiotropic developmental defects and preferential down-regulation of multiple G2/M-specific genes in *Arabidopsis* . Plant Physiol. 157, 706–717. doi: 10.1104/pp.111.180836 21862669 PMC3192584

[B16] Hamada T. (2014). Microtubule organization and microtubule-associated proteins in plant cells. Int. Rev. Cell Mol. Biol. 312, 1–52. doi: 10.1016/B978-0-12-800178-3.00001-4 25262237

[B17] Herrmann A. Livanos P. Lipka E. Gadeyne A. Hauser M. T. Van Damme D.. (2018). Dual localized kinesin-12 POK2 plays multiple roles during cell division and interacts with MAP65-3. EMBO Rep. 19, e46085. doi: 10.15252/embr.201846085 30002118 PMC6123660

[B18] Ho C. M. Hotta T. Guo F. Roberson R. W. Lee Y. R. Liu B. (2011a). Interaction of antiparallel microtubules in the phragmoplast is mediated by the microtubule-associated protein MAP65-3 in *Arabidopsis* . Plant Cell. 23, 2909–2923. doi: 10.1105/tpc.110.078204 21873565 PMC3180800

[B19] Ho C. M. Hotta T. Kong Z. Zeng C. J. Sun J. Lee Y. R.. (2011b). Augmin plays a critical role in organizing the spindle and phragmoplast microtubule arrays in *Arabidopsis* . Plant Cell. 23, 2606–2618. doi: 10.1105/tpc.111.086892 21750235 PMC3226208

[B20] Ho C. M. Lee Y. R. Kiyama L. D. Dinesh-Kumar S. P. Liu B. (2012). *Arabidopsis* microtubule-associated protein MAP65-3 cross-links antiparallel microtubules toward their plus ends in the phragmoplast *via* its distinct c-terminal microtubule binding domain. Plant Cell 24, 2071–2085. doi: 10.1105/tpc.111.092569 22570443 PMC3442588

[B21] Hotta T. Kong Z. Ho C. M. Zeng C. J. Horio T. Fong S.. (2012). Characterization of the *Arabidopsis* augmin complex uncovers its critical function in the assembly of the acentrosomal spindle and phragmoplast microtubule arrays. Plant Cell. 24, 1494–1509. doi: 10.1105/tpc.112.096610 22505726 PMC3398559

[B22] Ito M. (2005). Conservation and diversification of three-repeat myb transcription factors in plants. J. Plant Res. 118, 61–69. doi: 10.1007/s10265-005-0192-8 15703854

[B23] Janski N. Masoud K. Batzenschlager M. Herzog E. Evrard J. L. Houlné G.. (2012). The GCP3-interacting proteins GIP1 and GIP2 are required for γ-tubulin complex protein localization, spindle integrity, and chromosomal stability. Plant Cell. 24, 1171–1187. doi: 10.1105/tpc.111.094904 22427335 PMC3336128

[B24] Jürgens G. (2005). Cytokinesis in higher plants. Annu. Rev. Plant Biol. 56, 281–299. doi: 10.1146/annurev.arplant.55.031903.141636 15862097

[B25] Kawamura E. Himmelspach R. Rashbrooke M. C. Whittington A. T. Gale K. R. Collings D. A.. (2006). MICROTUBULE ORGANIZATION 1 regulates structure and function of microtubule arrays during mitosis and cytokinesis in the *Arabidopsis* root. Plant Physiol. 140, 102–114. doi: 10.1104/pp.105.069989 16377747 PMC1326035

[B26] Kobayashi K. Suzuki T. Iwata E. Magyar Z. Bögre L. Ito M. (2015). MYB3Rs, plant homologs of myb oncoproteins, control cell cycle-regulated transcription and form DREAM-like complexes. Transcription 6, 106–111. doi: 10.1080/21541264.2015.1109746 26556011 PMC4802795

[B27] Komaki S. Abe T. Coutuer S. Inzé D. Russinova E. Hashimoto T. (2010). Nuclear-localized subtype of end-binding 1 protein regulates spindle organization in *Arabidopsis* . J. Cell Sci. 123, 451–459. doi: 10.1242/jcs.062703 20067996

[B28] Kong Z. Hotta T. Lee Y. R. Horio T. Liu B. (2010). The γ-tubulin complex protein GCP4 is required for organizing functional microtubule arrays in *Arabidopsis thaliana* . Plant Cell. 22, 191–204. doi: 10.1105/tpc.109.071191 20118227 PMC2828712

[B29] Lee Y. R. Li Y. Liu B. (2007). Two *Arabidopsis* phragmoplast-associated kinesins play a critical role in cytokinesis during male gametogenesis. Plant Cell. 19, 2595–2605. doi: 10.1105/tpc.107.050716 17720869 PMC2002617

[B30] Lee Y. R. Liu B. (2000). Identification of a phragmoplast-associated kinesin-related protein in higher plants. Curr. Biol. 10, 797–800. doi: 10.1016/S0960-9822(00)00564-9 10898978

[B31] Lee Y. R. Liu B. (2019). Microtubule nucleation for the assembly of acentrosomal microtubule arrays in plant cells. New Phytol. 222, 1705–1718. doi: 10.1111/nph.15705 30681146

[B32] Li M. Li H. (2003). A simple and highly efficient *Agrobacterium*-mediated rice transformation system. Shi Yan Sheng Wu Xue Bao 36, 289–294. 14574993

[B33] Li H. Sun B. Sasabe M. Deng X. Machida Y. Lin H.. (2017). *Arabidopsis* MAP65-4 plays a role in phragmoplast microtubule organization and marks the cortical cell division site. New Phytol. 215, 187–201. doi: 10.1111/nph.14532 28370001

[B34] Lloyd C. (2011). Plant cytokinesis: Circles within circles. Curr. Biol. 21, 926–927. doi: 10.1016/j.cub.2011.10.018 22115462

[B35] Lucas J. R. Shaw S. L. (2012). MAP65-1 and MAP65-2 promote cell proliferation and axial growth in *Arabidopsis* roots. Plant J. 71, 454–463. doi: 10.1111/j.1365-313X.2012.05002.x 22443289

[B36] Ma Q. Dai X. Xu Y. Guo J. Liu Y. Chen N.. (2009). Enhanced tolerance to chilling stress in *OsMYB3R-2* transgenic rice is mediated by alteration in cell cycle and ectopic expression of stress genes. Plant Physiol. 150, 244–256. doi: 10.1104/pp.108.133454 19279197 PMC2675741

[B37] Magyar Z. Bögre L. Ito M. (2016). DREAMs make plant cells to cycle or to become quiescent. Curr. Opin. Plant Biol. 34, 100–106. doi: 10.1016/j.pbi.2016.10.002 27816815

[B38] Ma D. Han R. (2020). Microtubule organization defects in *Arabidopsis thaliana* . Plant Biol. (Stuttg) 22, 971–980. doi: 10.1111/plb.13114 32215997

[B39] McMichael C. M. Bednarek S. Y. (2013). Cytoskeletal and membrane dynamics during higher plant cytokinesis. New Phytol. 197, 1039–1057. doi: 10.1111/nph.12122 23343343

[B40] Müller S. Fuchs E. Ovecka M. Wysocka-Diller J. Benfey P. N. Hauser M. T. (2002). Two new loci, *PLEIADE* and *HYADE*, implicate organ-specific regulation of cytokinesis in *Arabidopsis* . Plant Physiol. 130, 312–324. doi: 10.1104/pp.004416 12226511 PMC166564

[B41] Müller S. Jürgens G. (2016). Plant cytokinesis-no ring, no constriction but centrifugal construction of the partitioning membrane. Semin. Cell Dev. Biol. 53, 10–18. doi: 10.1016/j.semcdb.2015.10.037 26529278

[B42] Müller S. Smertenko A. Wagner V. Heinrich M. Hussey P. J. Hauser M. T. (2004). The plant microtubule-associated protein AtMAP65-3/PLE is essential for cytokinetic phragmoplast function. Curr. Biol. 14, 412–417. doi: 10.1016/j.cub.2004.02.032 15028217 PMC2867789

[B43] Murata T. Sano T. Sasabe M. Nonaka S. Higashiyama T. Hasezawa S.. (2013). Mechanism of microtubule array expansion in the cytokinetic phragmoplast. Nat. Commun. 4, 1967. doi: 10.1038/ncomms2967 23770826 PMC3709505

[B44] Nakagawa T. Kurose T. Hino T. Tanaka K. Kawamukai M. Niwa Y.. (2007). Development of series of gateway binary vectors, pGWBs, for realizing efficient construction of fusion genes for plant transformation. J. Biosci. Bioeng. 104, 34–41. doi: 10.1263/jbb.104.34 17697981

[B45] Park M. Touihri S. Müller I. Mayer U. Jürgens G. (2012). Sec1/Munc18 protein stabilizes fusion-competent syntaxin for membrane fusion in *Arabidopsis* cytokinesis. Dev. Cell. 22, 989–1000. doi: 10.1016/j.devcel.2012.03.002 22595672

[B46] Sasabe M. Kosetsu K. Hidaka M. Murase A. Machida Y. (2011). *Arabidopsis thaliana* MAP65-1 and MAP65-2 function redundantly with MAP65-3/PLEIADE in cytokinesis downstream of MPK4. Plant Signal Behav. 6, 743–747. doi: 10.4161/psb.6.5.15146 21455028 PMC3172854

[B47] Sasabe M. Machida Y. (2012). Regulation of organization and function of microtubules by the mitogen-activated protein kinase cascade during plant cytokinesis. Cytoskeleton (Hoboken) 69, 913–918. doi: 10.1002/cm.21072 23027702

[B48] Sasabe M. Soyano T. Takahashi Y. Sonobe S. Igarashi H. Itoh T. J.. (2006). Phosphorylation of NtMAP65-1 by a MAP kinase down-regulates its activity of microtubule bundling and stimulates progression of cytokinesis of tobacco cells. Genes Dev. 20, 1004–1014. doi: 10.1101/gad.1408106 16598040 PMC1472297

[B49] Sedbrook J. C. Ehrhardt D. W. Fisher S. E. Scheible W. R. Somerville C. R. (2004). The *Arabidopsis sku6/spiral1* gene encodes a plus end-localized microtubule-interacting protein involved in directional cell expansion. Plant Cell. 16, 1506–1520. doi: 10.1105/tpc.020644 15155883 PMC490042

[B50] Smertenko A. Assaad F. Baluška F. Bezanilla M. Buschmann H. Drakakaki G.. (2017). Plant cytokinesis: Terminology for structures and processes. Trends Cell Biol. 27, 885–894. doi: 10.1016/j.tcb.2017.08.008 28943203

[B51] Smertenko A. Hewitt S. L. Jacques C. N. Kacprzyk R. Liu Y. Marcec M. J.. (2018). Phragmoplast microtubule dynamics - a game of zones. J. Cell Sci. 131, jcs203331. doi: 10.1242/jcs.203331 29074579 PMC6518215

[B52] Smertenko A. P. Kaloriti D. Chang H. Y. Fiserova J. Opatrny Z. Hussey P. J. (2008). The c-terminal variable region specifies the dynamic properties of arabidopsis microtubule-associated protein MAP65 isotypes. Plant Cell. 20, 3346–3358. doi: 10.1105/tpc.108.063362 19060108 PMC2630438

[B53] Staehelin L. A. Hepler P. K. (1996). Cytokinesis in higher plants. Cell 84, 821–824. doi: 10.1016/S0092-8674(00)81060-0 8601305

[B54] Van Damme D. Van Poucke K. Boutant E. Ritzenthaler C. Inzé D. Geelen D. (2004). *In vivo* dynamics and differential microtubule-binding activities of MAP65 proteins. Plant Physiol. 136, 3956–3967. doi: 10.1104/pp.104.051623 15557096 PMC535828

[B55] Vandepoele K. Vlieghe K. Florquin K. Hennig L. Beemster G. T. Gruissem W.. (2005). Genome-wide identification of potential plant E2F target genes. Plant Physiol. 139, 316–328. doi: 10.1104/pp.105.066290 16126853 PMC1203381

[B56] Walczak C. E. Shaw S. L. (2010). A MAP for bundling microtubules. Cell 142, 364–367. doi: 10.1016/j.cell.2010.07.023 20691897

[B57] Wang S. Gu Y. Zebell S. G. Anderson L. K. Wang W. Mohan R.. (2014). A noncanonical role for the CKI-RB-E2F cell-cycle signaling pathway in plant effector-triggered immunity. Cell Host Microbe 16, 787–794. doi: 10.1016/j.chom.2014.10.005 25455564 PMC4282163

[B58] Xu J. Lee Y. J. Liu B. (2020). Establishment of a mitotic model system by transient expression of the d-type cyclin in differentiated leaf cells of tobacco (*Nicotiana benthamiana*). New Phytol. 226, 1213–1220. doi: 10.1111/nph.16309 31679162

[B59] Zhang H. Deng X. Sun B. Lee Van S. Kang Z. Lin H.. (2018). Role of the BUB3 protein in phragmoplast microtubule reorganization during cytokinesis. Nat. Plants 4, 485–494. doi: 10.1038/s41477-018-0192-z 29967519

[B60] Zhang D. Wadsworth P. Hepler P. K. (1990). Microtubule dynamics in living dividing plant cells: confocal imaging of microinjected fluorescent brain tubulin. Proc. Natl. Acad. Sci. U.S.A. 87, 8820–8824. doi: 10.1073/pnas.87.22.8820 11607116 PMC55051

