# Supplementary material for: Rice microtubule-associated protein OsMAP65-3.1, but not OsMAP65-3.2, plays a critical role in phragmoplast microtubule organization in cytokinesis
Source: Front Plant Sci. 2022 Oct 26;13:1030247. doi: 10.3389/fpls.2022.1030247 (PMC9643714; doi:10.3389/fpls.2022.1030247)
Supplement: Supplementary file 1 [file DataSheet_1.docx]

Supplementary Material

# Supplementary Figures and Tables

## Supplementary Figures


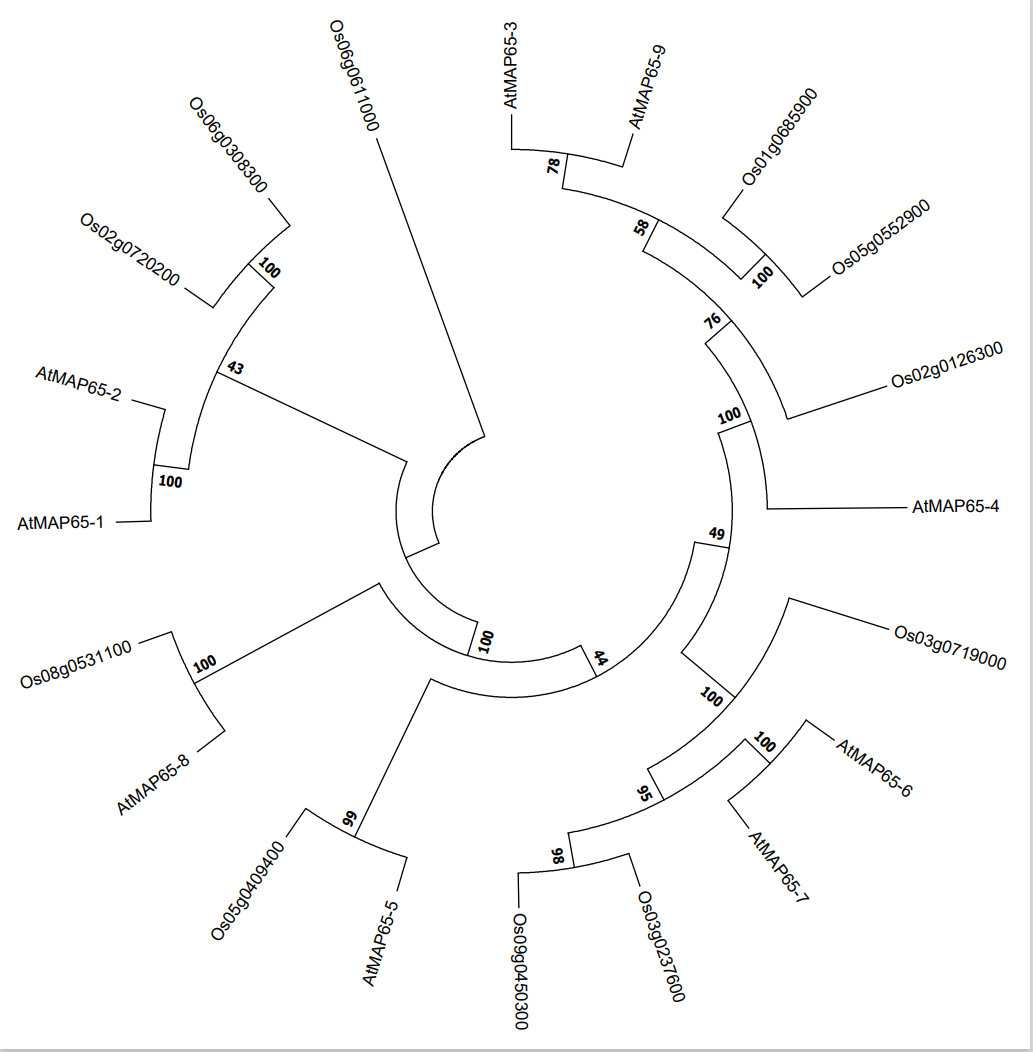


**Supplementary Figure 1.** Phylogenetic analysis of MAP65 gene family in Arabidopsis and rice. Bootstrap values indicate the divergence of each branch, and the scale shows branch length.


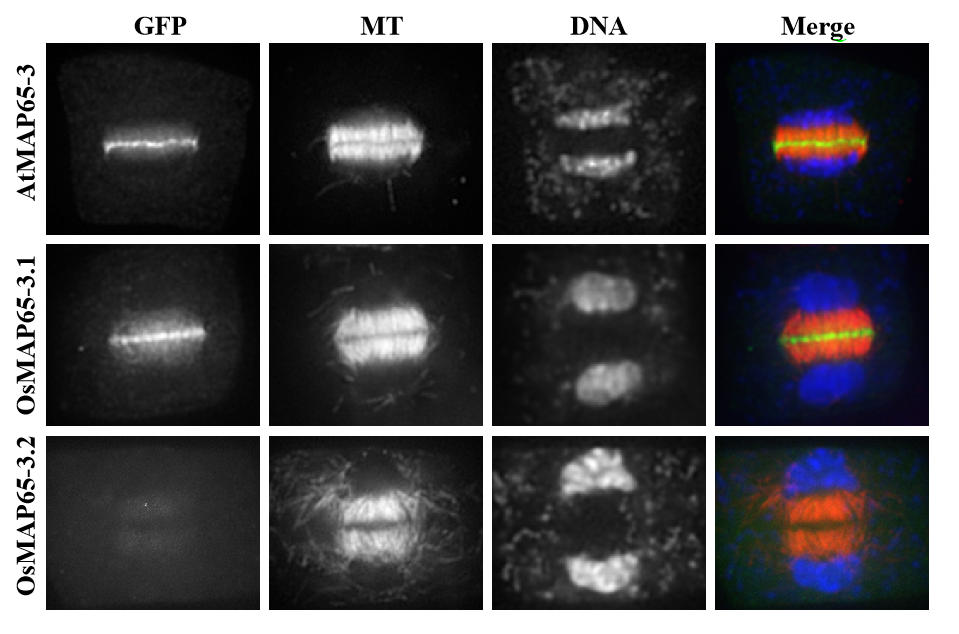


**Supplementary Figure 2.** Localization of AtMAP65-3-GFP, OsMAP65-3.1-GFP and OsMAP65-3.2-GFP with their own promoters in atmap65-3. The merged images have MAP65-3s-GFP detected by the anti-GFP antibody in green, MTs in red and DNA in blue.


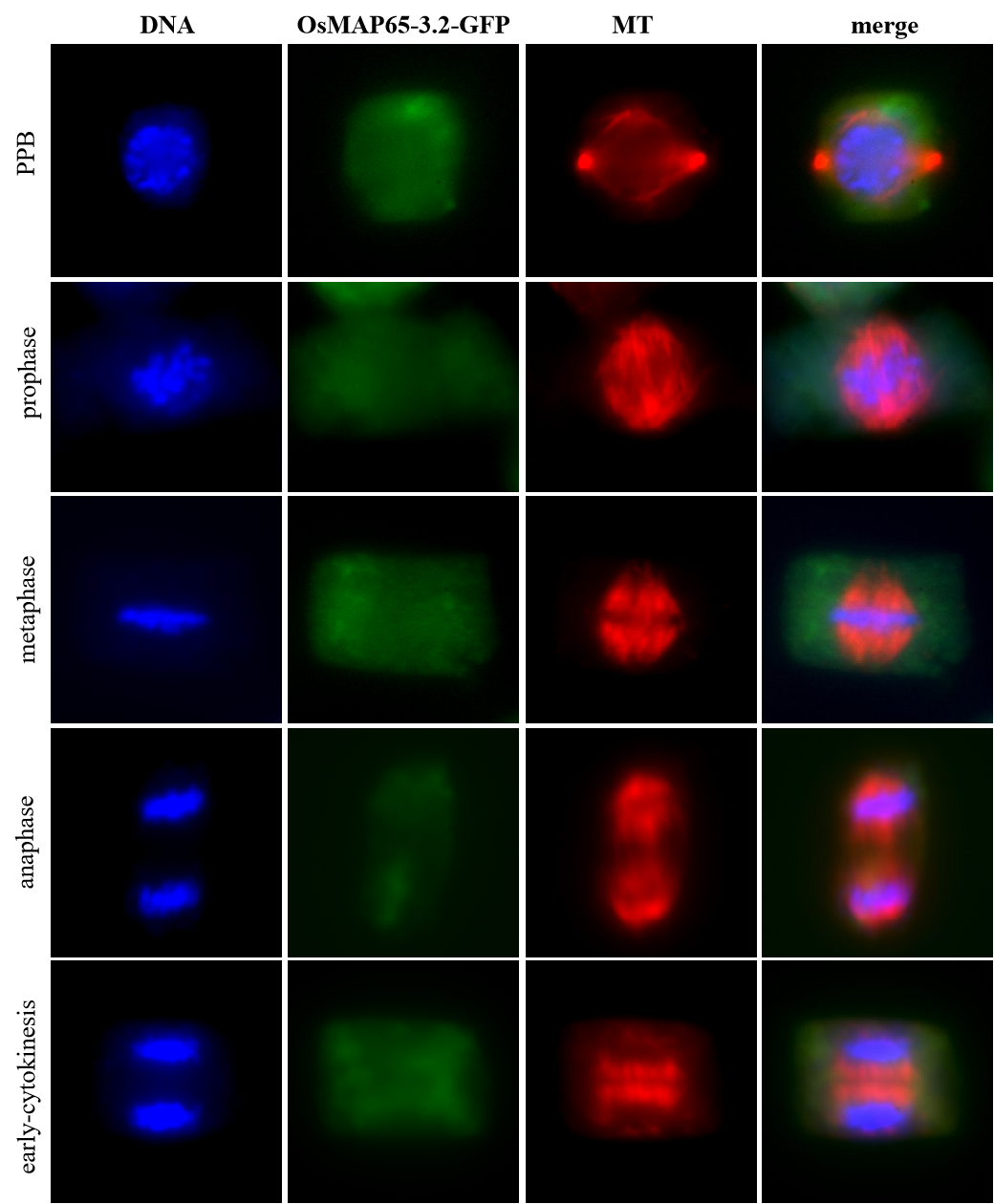


**Supplementary Figure 3.** Immunofluorescent localization of OsMAP65-3.2-GFP at MTs (PPB, spindles and phragmoplast) in prophase, metaphase, anaphase, telophase and cytokinesis in rice root meristematic cells.


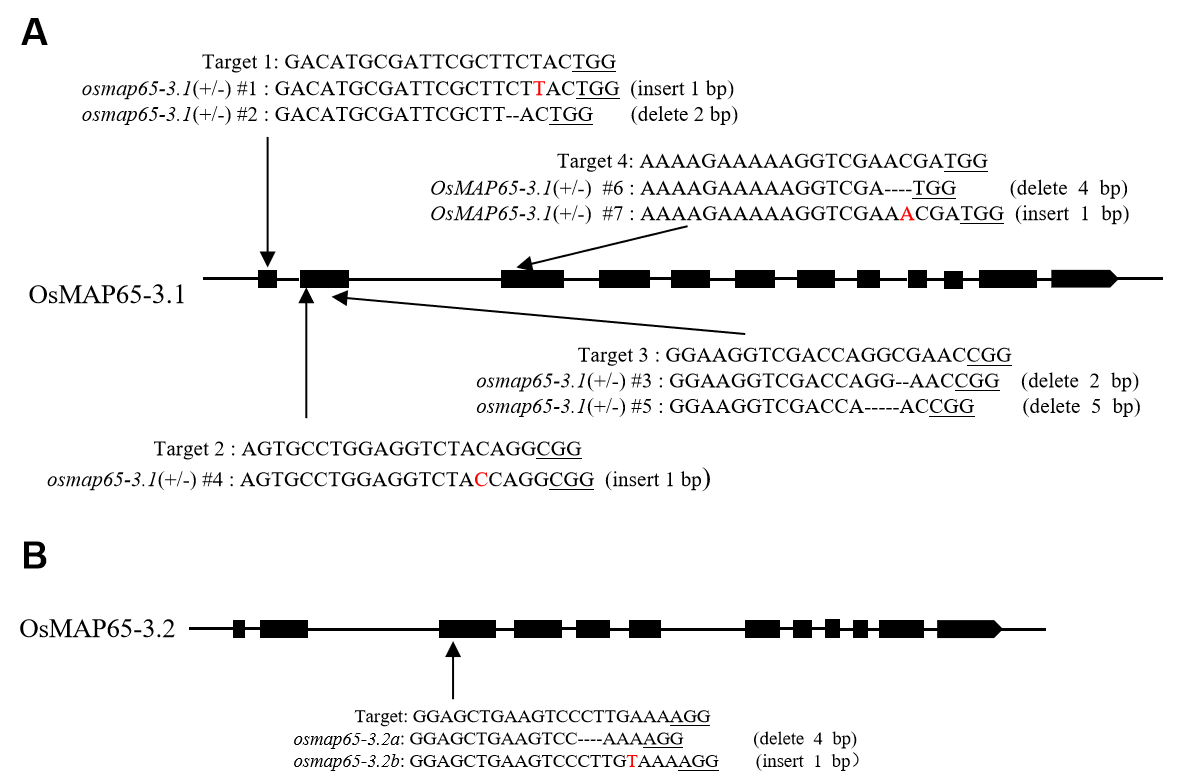


**Supplementary Figure 4.** Genotype of the *osmap65-3.1* and *osmap65-3.2* defect plants. **(A)** Seven *osmap65-3.1(+/-)* heterozygote generated by Crispr-cas9 system with 4 different target sites. **(B)** two independent osmap65-3.2 homozygote lines without vector fragment.


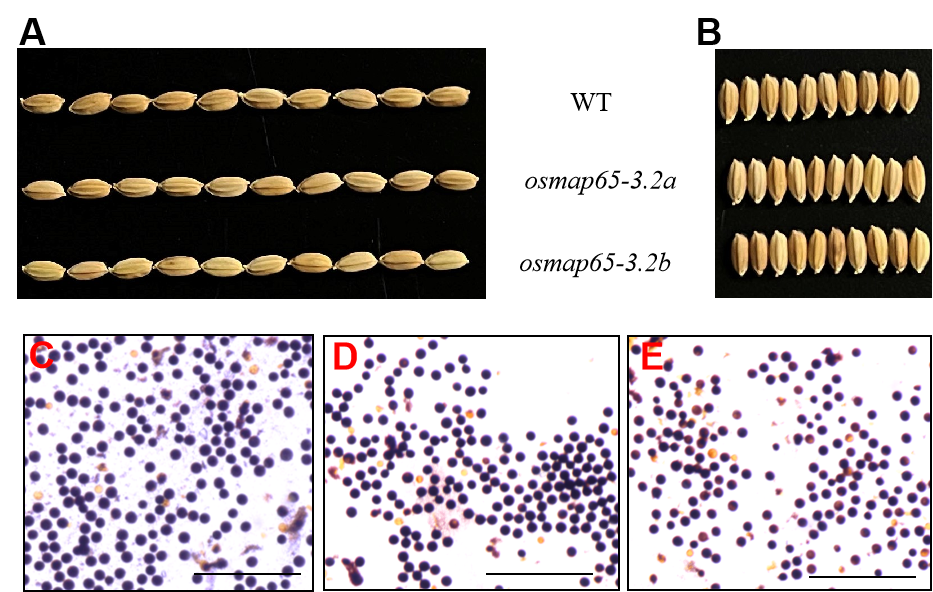


**Supplementary Figure 5.** Phenotypes of *osmap65-3.2* mutants. **(A-B)** Grain shape. **(C-E)** Pollen fertility of wild-type **(C)**, *osmap65-3.2a* **(D)** and *osmap65-3.2b* **(E)**.


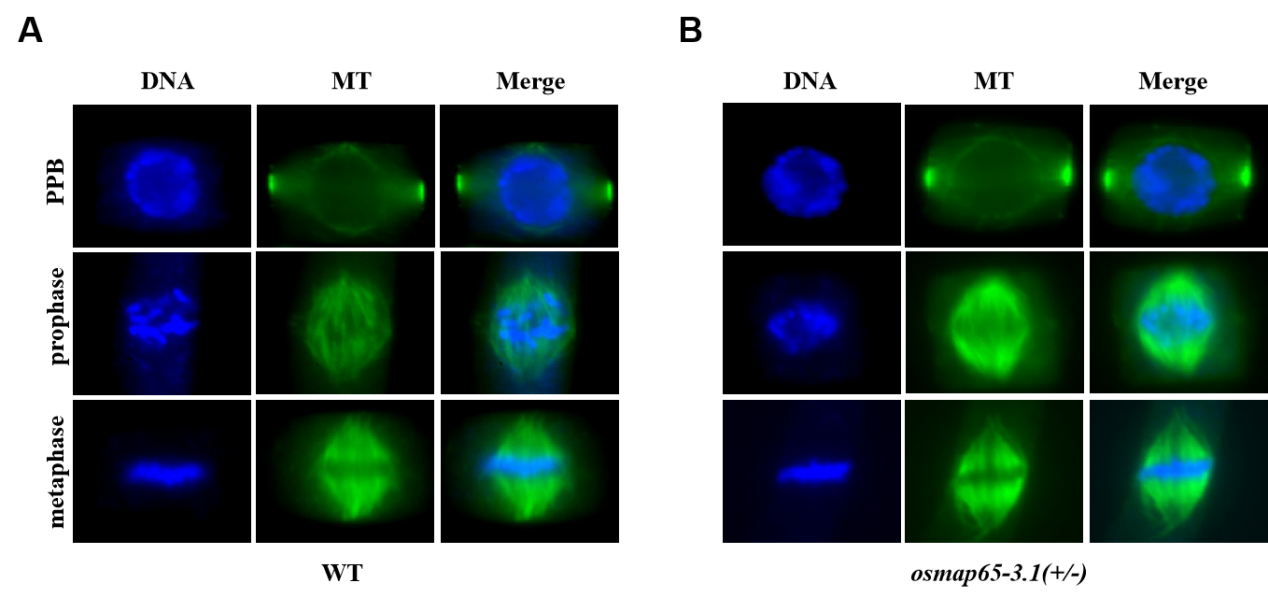


**Supplementary Figure 6.** PPB and spindles in *osmap65-3.1(+/-)* showed no obvious differences compared to wild-type. Wild-type cells **(A)** and *osmap65-3.1(+/-)* cells **(B)** at PPB, prophase and metaphase..


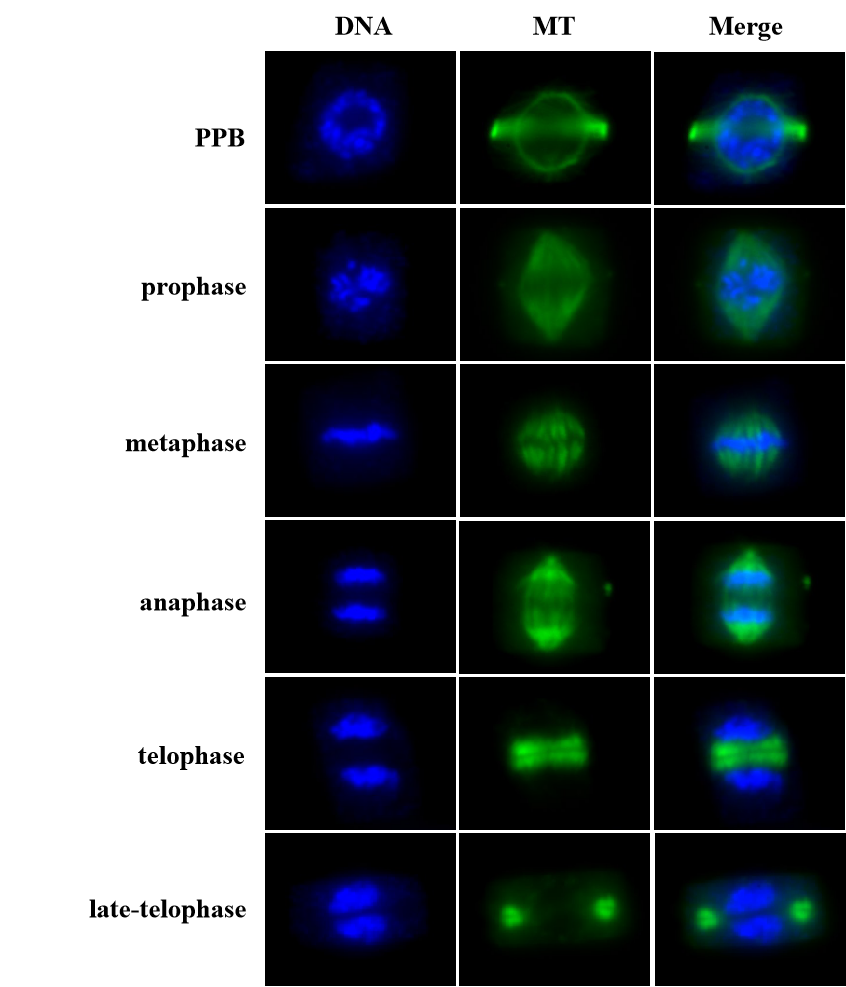


**Supplementary Figure 7.** *osmap65-3.2* have normal PPB, spindles and phragmoplast in root apical meristematic cells.


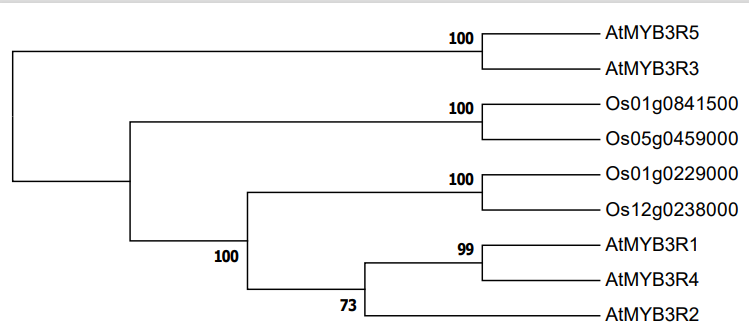


**Supplementary Figure 8.** Phylogenetic analysis of MYB3R transcription factors in *Arabidopsis* and rice. Bootstrap values indicate the divergence of each branch, and the scale shows branch length.


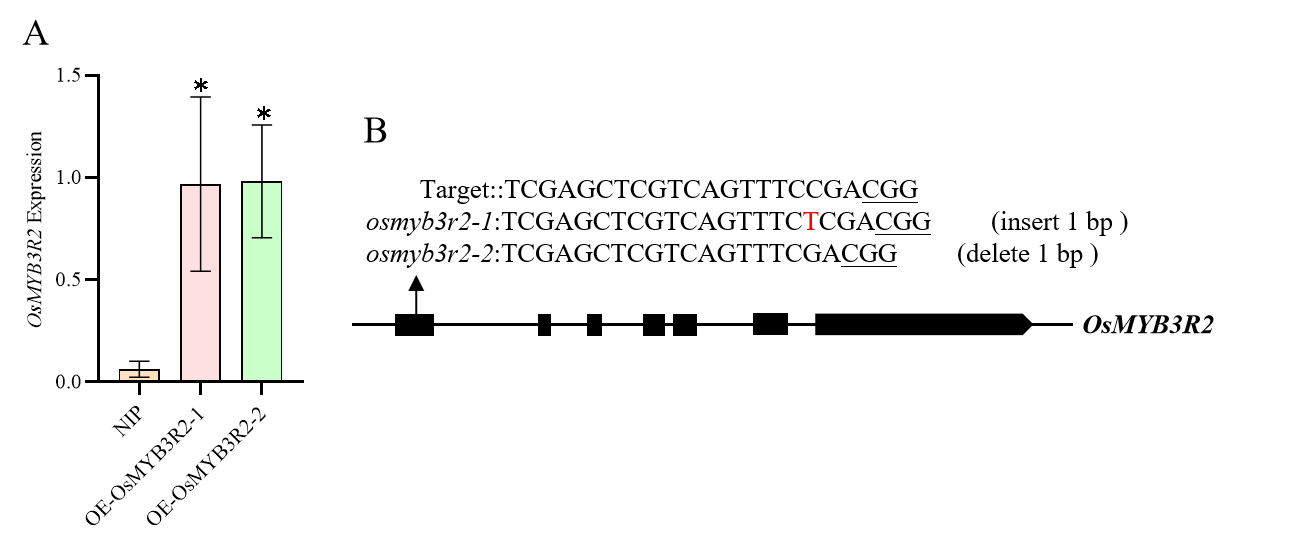


**Supplementary Figure 9.** Confirmation of over-expression and knockout plants of OsMYB3R2. **(A)** The transcription level of OsMYB3R2 in over-expression plants. **(B)** Two osmyb3r2 mutant lines generated by Crispr-cas9 system. *, P < 0.05 (Student’s t-test).


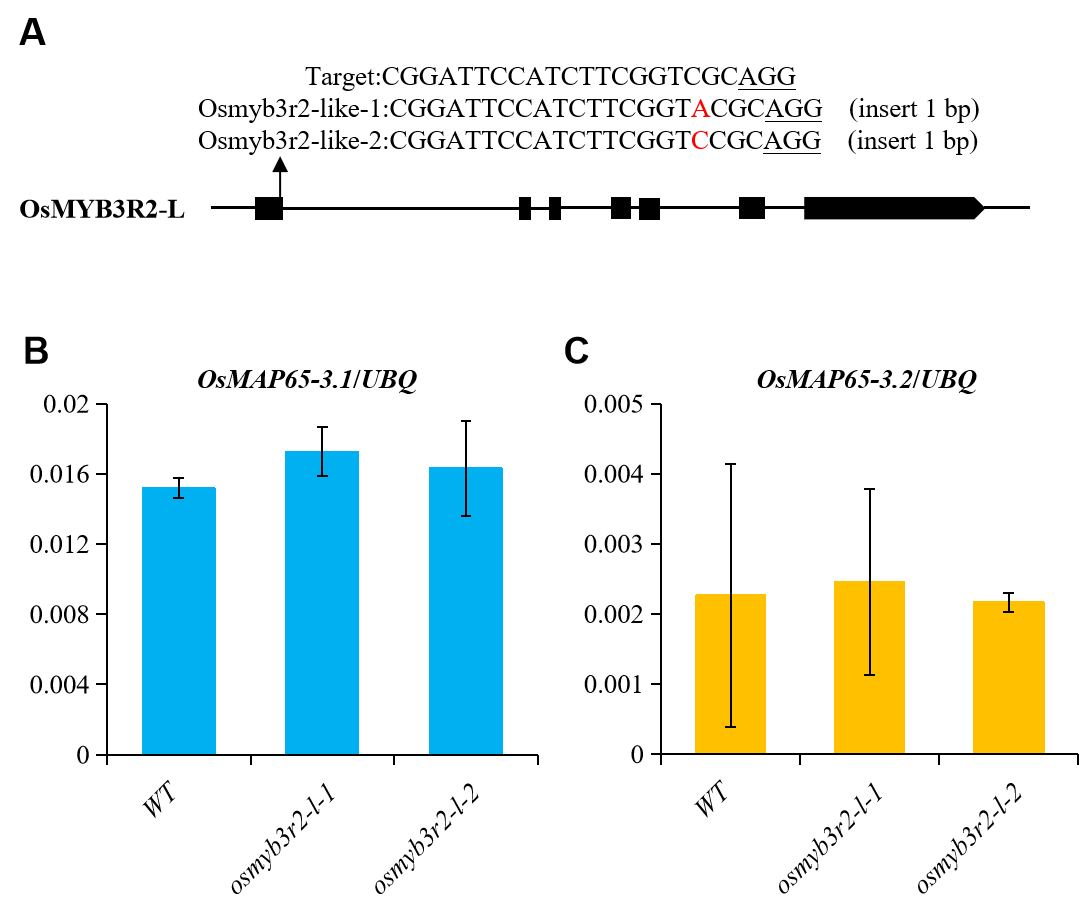


**Supplementary Figure 10.** The transcription level of *OsMAP65-3* genes in *osmyb3r2-l*. **(A)** Two *osmyb3r2-l* mutant lines generated by Crispr-cas9 system. **(B-C)** The expression of *OsMAP65-3.1* **(B)** and *OsMAP65-3.2* **(C)** in *osmyb3r2-l* mutant lines.

**Supplementary Table 1.** The sequences of the primers used for vectors construction

| Primers | Sequence | Using for |
| --- | --- | --- |
| AD-OsMAP65-3.1-F | CCATGGAGGCCAGTGAATTCATGAGTAGCGCGGTGAAGGACC | *OsMAP65-3.1* inserted to pGADT7 |
| AD-OsMAP65-3.1-R | AGCTCGAGCTCGATGGATCCTTAAACCATTTGCCTGGCCAGATAGATGGC |  |
| AD-OsMAP65-3.2-F | GCCATGGAGGCCAGTGAATTCATGAAGACCATATGCGGATCCCTC | *OsMAP65-3.2* inserted to pGADT7 |
| AD-OsMAP65-3.2-R | CAGCTCGAGCTCGATGGATCCTCAAGCCACTTGTGCGGCAAG |  |
| BD-OsMAP65-3.1-F | ATGGCCATGGAGGCCGAATTCATGAGTAGCGCGGTGAAGGACC | *OsMAP65-3.1* inserted to pGBKT7 |
| BD-OsMAP65-3.1-R | CGCTGCAGGTCGACGGATCCTTAAACCATTTGCCTGGCCAGATAGATGGC |  |
| BD-OsMAP65-3.2-F | ATGGCCATGGAGGCCGAATTCATGAAGACCATATGCGGATCCCTC | *OsMAP65-3.2* inserted to pGBKT7 |
| BD-OsMAP65-3.2-R | CCGCTGCAGGTCGACGGATCCTCAAGCCACTTGTGCGGCAAG |  |
| anti-OsMAP65-3.1-F | GCAGGTCGACTCTAGAGGATCCGTTTTAAAAAAGAAAGCAGAACTAGAAGAGCATAGAAG | knock-down of OsMAP65-3.1 |
| anti-OsMAP65-3.1-R | TCGCGAGCTCGGTACCAACCATTTGCCTGGCCAGATAGATGG |  |
| pENTR4-OsMAP65-3.1-F | GACAAGTTTGTACAAAAAAGCAGGCTTAGCACAGTTTGCATGTAAATCGCGAGAC | full length of *OsMAP65-3.1* and *OsMAP65-2* inserted to pENTR4 |
| pENTR4-OsMAP65-3.1-R | GACCACTTTGTACAAGAAAGCTGGGTTAACCATTTGCCTGGCCAGATAGATGGCGAGCCG |  |
| pENTR4-OsMAP65-3.2-F | GACAAGTTTGTACAAAAAAGCAGGCTTACTATATGCCCGTTCTGTAGCCAGGACG |  |
| pENTR4-OsMAP65-3.2-R | GACCACTTTGTACAAGAAAGCTGGGTTAGCCACTTGTGCGGCAAGATAGACTGC |  |
| 1300-ATP(OsMAP65-3.1)-F | TGACCATGATTACGAATTCTGATTCACAGTGAAACAAGCAAGAAGGGG | The CDS of *OsMAP65-3.1* or *OsMAP65-2* together with AtMAP65-3 promoter inserted to a motified pCAMBIA1300 |
| 1300-ATP(OsMAP65-3.1)-R | GCTACTCATTGCCATTTCGAAATGCTTAAGCCTG |  |
| 1300-OsMAP65-3.1(ATP)-F | GAAATGGCAATGAGTAGCGCGGTGAAGGACC |  |
| 1300-OsMAP65-3.1(ATP)-R | GGGTACCGAGCTCGAATTCTTAAACCATTTGCCTGGCCAGATAGATG |  |
| 1300-ATP(OsMAP65-3.2)-F | TGACCATGATTACGAATTCTGATTCACAGTGAAACAAGCAAGAAGGGG |  |
| 1300-ATP(OsMAP65-3.2)-R | GGTCTTCATTGCCATTTCGAAATGCTTAAGCCTG |  |
| 1300-OsMAP65-3.2(ATP)-F | GAAATGGCAATGAAGACCATATGCGGATCCCTC |  |
| 1300-OsMAP65-3.2(ATP)-R | GGGTACCGAGCTCGAATTCTCAAGCCACTTGTGCGGCAAG |  |
| 1305-OsMAP65-3.1P-F | GCTATGACCATGATTACGAATTCACAGTTTGCATGTAAATCGCGAGAC | The promoter of *OsMAP65-3.1* and *OsMAP65-2* inserted to pCAMBIA1305 |
| 1305-OsMAP65-3.1P-R | GAAATTTACCCTCAGATCTACCATCGCGAGGTGCGAAGAACAC |  |
| 1305-OsMAP65-3.2P-F | GCTATGACCATGATTACGAATTCTATATGCCCGTTCTGTAGCCAGGACG |  |
| 1305-OsMAP65-3.2P-R | GAAATTTACCCTCAGATCTACCATCGGGAGGAGGCGCTCCTTCGGATTGCC |  |
| 3.2Pro-PLACZi2u-2F | GCTATCATTTCCTTTGATATTGGATCGGAATTCAAAATTGATTTTAGGATTTTTCATTATAGTCAAC | The promoter of *OsMAP65-3.1* and *OsMAP65-2* inserted to pPLACZi2u |
| 3.2Pro-PLACZi2u-2R | AGAGTTTTATATACATACAGAGCACATGCCTCGAGGAAGAGAATTGTTCGAAACAACTTGAAAATGGGA |  |
| 3.1ProPLACZi2u-2F | GCTATCATTTCCTTTGATATTGGATCGGAATTCTTTTTTCTTCAAACCTTCTATTTTTCCGTCAC |  |
| 3.1Pro-PLACZi2u-2R | AGAGTTTTATATACATACAGAGCACATGCCTCGAGGGCAGAGAGACGTTAGCGAGGGAGCCTT |  |
| MYB3R2L-PB42AD-F | ATGATGTGCCAGATTATGCCTCTCCCGAATTCATGGGCGCGATGCCGGCGGTGAAGGTG | The CDS of OsMYB3R2 and OsMYB3R2L inserted to pPB42AD |
| MYB3R2L-PB42AD-R | CCAAACCTCTGGCGAAGAAGTCCAAAGCTTCTCGAGCAAATTAGGTTGGGTAGCATCCAACTTTG |  |
| MYB3R2-PB42AD-F | ATGATGTGCCAGATTATGCCTCTCCCGAATTCATGGGGGCCATGGCGATGGTGGAGCAGG |  |
| MYB3R2-PB42AD-R | CCAAACCTCTGGCGAAGAAGTCCAAAGCTTCTCGAGGGTTACATCCAAATTGGTTGTATGCGCAA |  |
| 1300S-MYB3R2L-F | TCGAGCTTTCGCGAGCTCGGTACCATGGGCGCGATGCCGG | Over-expression of OsMYB3R2 and OsMYB3R2L |
| 1300S-MYB3R2L-R | AGGTCGACTCTAGAGGATCCCAAATTAGGTTGGGTAGCATCCAACTTTGTCGTATGC |  |
| 1300S-MYB3R2-F | CGAGCTTTCGCGAGCTCGGTACCATGGGGGCCATGGCGATGGTGGAGCAGGAG |  |
| 1300S-MYB3R2-R | GGTCGACTCTAGAGGATCCGGTTACATCCAAATTGGTTGTATGCGCAAAGT |  |

**Supplementary Table 2.** The sequences of the oligonucleotides used for gene editing

| **Oligonucleotides** | **Sequence** | **Using for** |
| --- | --- | --- |
| CSMAP65-3.1-SgRNA1-F | CAGGACATGCGATTCGCTTCTAC | Knock out of *OsMAP65-3.1* |
| CSMAP65-3.1-SgRNA1-R | AACGTAGAAGCGAATCGCATGTC |  |
| CSMAP65-3.1-SgRNA2-F | CAGAGTGCCTGGAGGTCTACAGG |  |
| CSMAP65-3.1-SgRNA2-R | AACCCTGTAGACCTCCAGGCACT |  |
| CSMAP65-3.1-SgRNA3-F | CAGGGAAGGTCGACCAGGCGAAC |  |
| CSMAP65-3.1-SgRNA3-R | AACGTTCGCCTGGTCGACCTTCC |  |
| CSMAP65-3.1-SgRNA4-F | CAGAAAAGAAAAAGGTCGAACGA |  |
| CSMAP65-3.1-SgRNA4-R | AACTCGTTCGACCTTTTTCTTTT |  |
| CSMAP65-3.2-SgRNA-F | CAGGGAGCTGAAGTCCCTTGAAA | Knock out of *OsMAP65-3.2* |
| CSMAP65-3.2-SgRNA-R | AACTTTCAAGGGACTTCAGCTCC |  |
| CSMYB3R2L-SgRNA-F | CAGCGGATTCCATCTTCGGTCGC | Knock out of *OsMYB3R2L* |
| CSMYB3R2L-SgRNA-R | AACGCGACCGAAGATGGAATCCG |  |
| CSMYB3R2-SgRNA-F | CAGTCGAGCTCGTCAGTTTCCGA | Knock out of *OsMYB3R2* |
| CSMYB3R2-SgRNA-R | AACTCGGAAACTGACGAGCTCGA |  |

**Supplementary Table .** The sequences of the primers used for qPCR and CHIP-qPCR

| Primers | Forward sequence | Reverse sequence |
| --- | --- | --- |
| chip3.1P-1 | GCTTAATAAATTTGTCTCGCAGTTTAC | CTCTATGGCTGTGTTTAGTTCGTGTGCC |
| chip3.1P-2 | GAAATATTGCACTTGTAATACGTAGC | GTTTGCTAGCCAGGTGTTAAACGCGAA |
| chip3.1P-3 | CGCATAACCAGCTTATATTTTGTAGC | GTGTACTGACAGGTGGGGCCCATTGTTTG |
| chip3.1P-4 | GCTGCGGGGAGCCGGCCCAATAAAGGC | CTGTAGGGGGAGGGGAGGAGAGGCGGA |
| chip3.1P-5 | GACGAGACCGCGCCGAGCGGCGAAGAG | CTACCGCCGCCGCCGCAGCGCGCGTCAG |
| chip3.1P-6 | GAAGGACCAGCTTCACCAGATGTCGAC | CTCCACTGCCATGCCAGAACCAACAC |
| chip3.2P-1 | CCTTTTCTCCTTTTACCCCTAATAAGGAAC | CTATGCTTCATGGATCTGCATTATAAAG |
| chip3.2P-2 | CTTTATTATTTCCATATCCATGCTTTGG | GTTGAGAAGTTGACTATAATGAAAAATCC |
| chip3.2P-3 | CCGTAATACAGATACAAAATTTTTACC | GATTATGCTGAGTGGTGAGTAGTGAG |
| chip3.2P-4 | GAACAATTCTCTTCAATCCCTCATCATTTC | GAAACGAGGGCACCGCTGCGTCGTCCTC |
| chip3.2P-5 | GTGAACTCGAGGCCGTAGGCGTTACTG | GCCGCTCCTCGTGAGGACGATACCAC |
| chip3.2P-6 | GTTGATCGTGCCAACCCTAATTCTGAC | CGGGAGGAGGCGCTCCTTCGGATTGCC |
| RT-MYB3R2L | CTTCCTGGAAGGACAGACAATTCTATAAAAAAC | CTGTTTCAAACCATCACCAACAACTG |
| RT-MYB3R2 | CTGGAAGAAAATAGCCGAATGTTTTCC | GATCATCTTCCTCTTGAGTCCAAGGAC |
| RT-OsMAP65-3.1 | CTTCAGAAGGAGAAGAGCGATCGGC | GGGTGCACCTCATATACTGTTTGC |
| RT-OsMAP65-3.2 | GGATGCAGAAGCTTCAAGATCTTGC | GCCTCTGAAGCAGCAATATTACATG |
| RT-UBQ | ACCACTTCGACCGCCACTACT | ACGCCTAAGCCTGCTGGTT |

**Supplementary Table 4.** Homology analysis of six proteins in MAP65-3/MAP65-4/MAP65-9 clade

| **rice genes** | **arabidopsis genes** | **full length** | **N terminal** | **MTB1 domain** | **MTB2 domain** |
| --- | --- | --- | --- | --- | --- |
| Os01g0685900 | AtMAP65-3 | 55.35% | 61.01% | 64.90% | 41.70% |
|  | AtMAP65-4 | 45.96% | 45.05% | 68.39% | 29.53% |
|  | AtMAP65-9 | 49.73% | 48.68% | 59.35% | none |
| Os05g0552900 | AtMAP65-3 | 53.18% | 60.67% | 65.58% | 34.55% |
|  | AtMAP65-4 | 45.43% | 44.82% | 65.56% | none |
|  | AtMAP65-9 | 52.46% | 50.00% | 59.09% | 55.56% |
| Os02g0126300 | AtMAP65-3 | 48.31% | 49.10% | 54.90% | none |
|  | AtMAP65-4 | 41.94% | 39.33% | 55.56% | none |
|  | AtMAP65-9 | 43.75% | 42.31% | 56.38% | none |

**Supplementary Table 5.** The main agronomic traits of *osmap65-3.2* mutants

|  | **Wild-type** | ***osmap65-3.2a*** | ***osmap65-3.2b*** |
| --- | --- | --- | --- |
| Plant height (cm) | 80.04 ± 3.809 | 73.75 ± 1.924* | 71.90 ± 3.552* |
| Panicle number | 14.60 ± 3.406 | 15.23 ± 3.444 | 14.74 ± 3.942 |
| Panicle length (cm) | 20.13 ± 1.479 | 17.06 ± 1.039* | 17.17 ± 0.978* |
| Primary branches | 8.07 ± 0.868 | 8.13 ± 1.655 | 8.00 ± 0.831 |
| Secondary branches | 20.03 ± 3.134 | 17.48 ± 4.423 | 18.59 ± 2.899 |
| Seed-setting (%) | 70.59 ± 7.095 | 70.88 ± 8.091 | 74.28 ± 8.994* |
| Grain length (mm) | 7.66 ± 0.192 | 7.592 ± 0.180 | 7.64 ± 0.181 |
| Grain width (mm) | 3.33 ± 0.115 | 3.31 ± 0.132 | 3.286 ± 0.107 |
| Grain thickness (mm) | 2.28 ± 0.065 | 2.29 ± 0.082 | 2.28 ± 0.078 |
| Thousand grain weight (g) | 26.29 ± 0.612 | 27.28 ± 1.21 | 25.26 ± 0.784 |

Note: Data are Mean ±SD (n = 5). *, P < 0.05 (Student’s t-test).

**Supplementary Table 6.** The common cis-acting elements in promoters of *AtMAP65-3*, *OsMAP65-3.1* and *OsMAP65-3.2*

| **Name** | **Sequence** | ***AtMAP65-3* promoter**  **1039 bp** | ***OsMAP65-3.1* promoter**  **841 bp** | ***OsMAP65-3.2* promoter**  **476 bp** |
| --- | --- | --- | --- | --- |
| CAAT-box | CAAT | -160~-157, -360~-357,  -419~-416, -639~-636, | -33~-30, -81~-78,  -95~-92, -127~-124,  -193~-190, -428~-425 | -11~-8, -135~-132,  -400~-397 |
| STRE | AGGGG | -1011~-1015 | ` |  |
| **MSA** | **aacgg** | **-99~-95** | **-234~-230** |  |
| TATA-box | TATA | -298~-295, -341~-338,  -370~-367, -414~-411,  -416~-413, -461~-458,  -469~-466, -598~-595,  -614~-611, -646~-643,  -673~-670, -751~-748,  -925~-922 | -266~-263, -365~-362,  -367~-364, -595~-592,  -817~-814 | -93~-90, -292~-289,  -350~-347 |
| ARE | AAACCA | -364~-359, -396~-391,  -872~-867 |  |  |
| LTR | CCGAAA | -208~-203 |  |  |
| GT1-motif | GGTTAA |  | -238~-233 |  |
| ABRE | ACGTG |  | -153~-149, -250~-246 |  |
| WRE3 | CCACCT |  | -184~-179 |  |
| CGTCA-motif | CGTCA |  | -767~-763 |  |
| MYB | TAACCA  CAACAG |  | -275~-270 | -195~-190 |
| TGACG-motif | TGACG |  | -724~-720 |  |
| TCCC-motif | TCTCCCT |  |  | -40~-34 |
